# Brain-environment alignment during movie watching predicts fluid intelligence and affective function in adulthood

**DOI:** 10.1016/j.neuroimage.2021.118177

**Published:** 2021-09

**Authors:** Raluca Petrican, Kim S. Graham, Andrew D. Lawrence

**Affiliations:** Cardiff University Brain Research Imaging Centre (CUBRIC), School of Psychology, Cardiff University, Maindy Road, Cardiff CF24 4HQ, United Kingdom

**Keywords:** *Event cognition*, *Functional networks*, *Dynamic connectivity*, *Fluid intelligence*, *Aging*, *Anxietybrain-environment alignment during movie watching predicts fluid intelligence and affective function in adulthood*

## Abstract

BOLD fMRI studies have provided compelling evidence that the human brain demonstrates substantial moment-to-moment fluctuations in both activity and functional connectivity (FC) patterns. While the role of brain signal variability in fostering cognitive adaptation to ongoing environmental demands is well-documented, the relevance of moment-to-moment changes in FC patterns is still debated. Here, we adopt a graph theoretical approach in order to shed light on the cognitive-affective implications of FC variability and associated profiles of functional network communication in adulthood. Our goal is to identify brain communication pathways underlying FC reconfiguration at multiple timescales, thereby improving understanding of how faster perceptually bound versus slower conceptual processes shape neural tuning to the dynamics of the external world and, thus, indirectly, mold affective and cognitive responding to the environment. To this end, we used neuroimaging and behavioural data collected during movie watching by the Cambridge Center for Ageing and Neuroscience (*N* = 642, 326 women) and the Human Connectome Project (*N* = 176, 106 women). FC variability evoked by changes to both the concrete perceptual and the more abstract conceptual representation of an ongoing situation increased from young to older adulthood. However, coupling between variability in FC patterns and concrete environmental features was stronger at younger ages. FC variability (both moment-to-moment/concrete featural and abstract conceptual boundary-evoked) was associated with age-distinct profiles of network communication, specifically, greater functional integration of the default mode network in older adulthood, but greater informational flow across neural networks implicated in environmentally driven attention and control (cingulo-opercular, salience, ventral attention) in younger adulthood. Whole-brain communication pathways anchored in default mode regions relevant to episodic and semantic context creation (i.e., angular and middle temporal gyri) supported FC reconfiguration in response to changes in the conceptual representation of an ongoing situation (i.e., narrative event boundaries), as well as stronger coupling between moment-to-moment fluctuations in FC and concrete environmental features. Fluid intelligence/abstract reasoning was directly linked to levels of brain-environment alignment, but only indirectly associated with levels of FC variability. Specifically, stronger coupling between moment-to-moment FC variability and concrete environmental features predicted poorer fluid intelligence and greater affectively driven environmental vigilance. Complementarily, across the adult lifespan, higher fluid (but not crystallised) intelligence was related to stronger expression of the network communication profile underlying momentary and event boundary-based FC variability during youth. Our results indicate that the adaptiveness of dynamic FC reconfiguration during naturalistic information processing changes across the lifespan due to the associated network communication profiles. Moreover, our findings on brain-environment alignment complement the existing literature on the beneficial consequences of modulating brain signal variability in response to environmental complexity. Specifically, they imply that coupling between moment-to-moment FC variability and concrete environmental features may index a bias towards perceptually-bound, rather than conceptual processing, which hinders affective functioning and strategic cognitive engagement with the external environment.

## Highlights

• Functional brain connectivity (FC) patterns vary with changes in the environment.
• Adult FC variability is linked to age-specific network communication profiles.
• Across adulthood, the younger network interaction profile predicts higher fluid IQ.
• Yoked FC-concrete environmental changes predict poorer fluid IQ and anxiety.
• Brain areas linked to episodic memory underpin FC changes at multiple timescales.

## 1 Introduction

BOLD fMRI studies testify to the importance of acknowledging the highly dynamic nature of the human brain in order to better understand its contribution to optimal lifespan functioning (Garrett et al., 2015, 2017, 2020; Grady and Garrett, 2014; Poldrack and Shine, 2018). A substantial portion of this research has focused on moment-to-moment variability (i.e., within-individual standard deviation) in BOLD signal and provided compelling evidence that its flexible modulation based on current task requirements (e.g., external vs. internal attentional focus, difficulty level) is key to superior cognitive performance across all ages (Garrett et al., 2020; Garrett et al., 2013a Grady and Garrett, 2018; Roberts et al., 2020). The interpretation is that BOLD signal variability reflects neural flexibility, specifically, the capacity of a brain to tune into the dynamics of the external world and respond in a differentiated manner to a wide range of environmental stimuli (Garrett et al., 2020; Grady and Garrett, 2018; Padmanabhan and Urban, 2010).

Moment-to-moment fluctuations are observed not only in the BOLD signal arising from individual brain regions, but also in the *correlations* of BOLD signals originating from different brain regions (Poldrack and Shine, 2018). However, the relevance of such time-varying functional connectivity (FC) patterns is still debated, with some studies underscoring their role in maturation and learning, whereas others point to their association with poorer attentional performance and accelerated cognitive aging in later life (Bassett et al., 2011, 2013; Fong et al., 2019; Hutchison and Morton, 2015; Mujica-Parodi et al., 2020).

Here, we seek to shed further light on this issue by probing whether, similar to BOLD signal variability, FC variability can foster brain-environment alignment, that is, adaptation of the brain's functional organization to ongoing changes in the external world (Cohen, 2018). To characterize fluctuations in whole-brain FC patterns at short timescales (~40 s), we adopted a combined sliding window-graph theoretical method due to its proven sensitivity to detecting behaviourally yoked FC reconfiguration (e.g., during learning, Bassett et al., 2011, 2013). In this approach, region-to-region FC over fixed-length temporal segments (i.e., “windows”) is used to break down the whole brain network into non-overlapping functional communities or clusters (Cohen, 2018; Poldrack and Shine, 2018). Roughly speaking, window-to-window or dynamic FC reconfiguration is then estimated as the percentage of changed pairwise functional clusterings across all brain regions.

To investigate the real-life adaptiveness of dynamic FC reconfiguration, we applied the aforementioned sliding window-graph theoretical method to functional neuroimaging data collected during movie watching, a naturalistic cognition paradigm well-suited for characterizing individual differences in continuous information processing (Bottenhorn et al., 2019; Demirtaş et al., 2019; Finn and Bandettini, 2021; Geerligs et al., 2018; Simony et al., 2016; Sonkusare et al., 2019). In using this paradigm, we capitalize on evidence that the human mind readily breaks down the uninterrupted influx of environmental information into meaningful units, which it uses to predict and encode ongoing experiences (Baldwin and Kosie, 2020; Kurby & Zacks, 2008). Such segmentation processes unfold at multiple timescales and give rise to a nested experiential hierarchy, which spans relatively frequent and readily processed changes to an observer's concrete representation of the immediate environment (e.g., object presence/absence) to relatively sporadic and slower processed changes to an observer's more abstract, conceptual representation of an ongoing situation, the so-called event model (cf. Event Segmentation Theory, Zacks, 2020). The movie watching paradigm thus provides a naturalistic framework for identifying meaningful environmental changes in response to which FC reconfiguration may be adaptive.

Applying insights from the literature on BOLD signal variability, we thus sought to elucidate whether FC reconfiguration associated with (1) moment-to-moment concrete *featural* fluctuations in the immediate environment (e.g., object presence/absence) and (2) more sporadic and abstract conceptual changes in the *relationships* among the various components of an ongoing situation (e.g., human actors, inanimate objects, spatial layout) could reflect a brain's capacity to respond in a more differentiated manner and, thus, better adapt to the external world (McDonnell and Ward, 2011). We conceptualize FC reconfiguration in response to changes in the concrete featural versus the more abstract relational representation of the current “state of play” as reflecting complementary routes to achieving optimal adaptation to the external milieu. The former is likely to indicate brain sensitivity to the perceptual richness of the immediate environment. Consequently, it seems the closest analogue of the increased BOLD signal variability which is observed in more complex perceptual environments and contributes to superior cognitive functioning (Garrett et al., 2020). Complementarily, FC reconfiguration linked to changes in the more abstract relational representation of an ongoing situation, the so-called event boundaries (Zacks, 2020), likely reflects brain sensitivity to the fit between one's current model of the world and informational influx from the immediate perceptual reality. Consequently, this type of FC variability could presumably support top-down driven, strategic adaptation to the external environment.

To characterize the contribution of FC reconfiguration to optimal performance across the adult lifespan, we included indices of affective and cognitive functioning. The latter was gauged with a test of fluid intelligence, a mental capacity reliant on well-differentiated perceptual representations of the external world, which supports flexible updating of abstract event representations and strategic adjustment to novel environments (Cattell, 1971; Colzato et al., 2006; Duncan et al., 2020, 2017). Affective functioning was measured with depression and anxiety scales because the two capture complementary biases in attentional engagement with the external milieu (Brewin et al., 2010; Sherrill et al., 2019; Sylverster et al., 2012). Specifically, depression has been associated with a bias towards abstract, internally generated information and disengagement from the immediate external milieu, whereas anxiety is reportedly typified by a bias towards concrete, external information and hypervigilance to perceptual environmental stimuli, even if irrelevant to ongoing task demands (Belzung et al., 2015; Bishop et al., 2004; Hermans et al., 2014; Moser et al., 2012; Petrican et al., 2015). Using these measures and the literature on BOLD signal variability as a conceptual background, we pursued four lines of inquiry regarding the relevance of FC variability to cognitive and affective functioning in adulthood, which we detail below.

### 1.1 Fluid intelligence and FC variability across adulthood

Brain signal variability has been shown to make a substantial contribution to superior mental performance in adulthood, while its aging-related decline has been demonstrated to account for some of the cognitive deficits observed in older age, particularly those involved in responding adaptively to the external environment (Garrett et al., 2010; Garrett et al., 2020). Consequently, our first objective was to establish whether (1) levels of moment-to-moment and event boundary-based FC reconfiguration similarly underpin superior cognitive functioning in adulthood, and whether (2) their fluctuations across the lifespan could account for deficits in fluid intelligence, a cognitive ability that tends to decline steadily from late adolescence onwards (McArdle et al., 2002).

If either type of FC variability reflects neural flexibility, then we would expect it to be positively linked to fluid intelligence, with its decline in older adulthood predicting fluid intelligence decrements, in line with prior findings on brain signal variability and adaptive responding to the external environment (Garrett et al., 2010, 2013a). If, on the other hand, greater FC reconfiguration during movie watching is an index of underlying architectural instability and, thus, deficient neural functioning, as it has been reported for resting state and experimental task paradigms (cf. Fong et al., 2019; Hilger et al., 2020; Mujica-Parodi et al., 2020), then we would expect it to be negatively linked to fluid intelligence and increase with age, with its increments predicting fluid intelligence decrements. To probe the specificity of any observed effects, we included a crystallized intelligence measure, which, as an index of accumulated knowledge, was not expected to be linked to our presumed indices of neural flexibility (i.e., the two types of FC variability).

### 1.2 Fluid intelligence and brain network interactions underpinning FC variability across adulthood

Studies on brain signal variability underscore the adaptiveness of activity fluctuations in functionally specific regions and networks, most widely, those involved in attending to the external world (visual, salience [SAL], dorsal attention [DAN], Garrett et al., 2020; Grady and Garrett, 2018). Similarly, literature on adaptive FC reconfiguration during learning highlights its association with greater functional integration of systems involved in external attention and environmentally driven control (SAL, DAN, cingulo-opercular [CON]), but weaker integration of these systems with the network generally implicated in self-generated cognition (default mode network, [DMN]) (Finc et al., 2020). These findings thus raise the possibility that it may not be levels of FC variability per se, but rather the associated network communication profiles which determine their adaptiveness during real-life information processing. Hence, our second objective was to identify the network interaction patterns which underpin moment-to-moment and event boundary-based FC reconfiguration and predict superior fluid intelligence across the adult lifespan.

Environmentally oriented control systems (i.e., CON) play a key role in fluid intelligence (Barbey, 2018; Duncan et al., 2020). However, with age, external processing networks (i.e., CON, SAL, DAN, ventral attention [VAN], perceptual systems) show declining modulation of brain signal variability, while the functional dominance of systems implicated in internal cognition increases (Garrett et al., 2013a; Grady and Garrett, 2018; Spreng and Turner, 2019). Based on these findings, we predicted that, across the adult lifespan, superior fluid intelligence would be associated with greater informational flow (i.e., integration) over external processing systems, a network interaction profile most likely to underpin to FC variability during younger adulthood.

### 1.3 Fluid intelligence and affective functioning: relevance of yoked FC and concrete environmental variability

Beyond mean variability levels, environmentally-driven modulation of brain signal variability, presumably a more sensitive index of neural differentiation, has been uniquely linked to superior cognitive functioning (Garrett et al., 2020). Consequently, our third objective was to probe whether strength of coupling between levels of FC reconfiguration and changes in concrete environmental features would predict fluid intelligence and affect-linked patterns of attentional engagement with the external milieu beyond mean levels of moment-to-moment FC variability.

If greater coupling between changes in FC patterns and concrete environmental features reflects greater attentional engagement with the external world, then we would expect it to be positively linked to anxiety, but negatively linked to depression (Belzung et al., 2015; Bishop et al., 2004; Hermans et al., 2014; Moser et al., 2012; Petrican et al., 2015). Furthermore, if, similar to brain signal variability (Garrett et al., 2020), such FC tuning to concrete environmental fluctuations fosters a more differentiated processing of the external milieu, then we would anticipate it to predict superior fluid intelligence (Duncan et al., 2020).

Our study focused on two concrete environmental feature types: (a) static (e.g., presence of objects/people, spatial layout characteristics), and (b) dynamic (i.e., natural phenomena, character actions). The former best approximate indices of perceptual complexity used to probe environmentally driven modulation of brain signal variability (Garrett et al., 2020) and, thus, they facilitate the most direct comparison with the respective literature. This is why static environmental features constituted the focus of our inquiry. Nonetheless, because dynamic environmental features are reportedly foundational to event segmentation processes from childhood onwards, we also explored the adaptiveness of their coupling with FC reconfiguration levels (Levine et al., 2019; Magliano and Zacks, 2011; Swallow et al., 2018).

### 1.4 Brain network interactions underpinning FC variability in response to concrete featural fluctuations and abstract event boundaries

There is compelling evidence that changes to the more abstract relational representation of an ongoing situation (i.e., event boundaries) are driven by fluctuations in concrete environmental features, such as those linked to spatial layout (Magliano and Zacks, 2011; Reagh et al., 2020). This raises the possibility that overlapping neural communication pathways underlie event perception processes across multiple timescales and levels of concreteness/abstraction. Identification of such circuits could provide valuable insight into how the brain tunes into the dynamics of the external world to support optimal information processing. Consequently, as an exploratory analysis, we sought to characterize the brain region-specific patterns of informational flow which sync up an observer's concrete perceptual representation of the immediate environment, as well as their more abstract conceptual model of an ongoing situation to changes in the external milieu.

To test our hypotheses, we employed data from two healthy adult samples: a lifespan sample from the Cambridge center for Ageing and Neuroscience (Cam-Can) and a young adult sample from the Human Connectome Project (HCP). The movies in the two datasets approximate well the range of naturalistic events (i.e., well-defined and temporally condensed story plot [Cam-Can] versus narratively looser and topic diverse movie assortment [HCP]) and, thus, enabled us to characterize the cognitive-affective correlates of FC dynamics across multiple timescales and levels of abstraction/concreteness. For the sake of concision, we combined methods and results across the two samples, but underscored any sample-specific features.

## 2 Methods

### 2.1 Participants

#### 2.1.1 Cam-Can

We included the largest number of participants from Stage II of the Cambridge Centre for Ageing and Neuroscience (Cam-Can) study with available fMRI data in the movie watching condition (*N* = 642, age range: 18–88 yrs [*M* = 54 yrs, SD = 19 yrs]).

The majority of participants (*N* = 589) were predominantly right-handed (handedness measure > 0). The sample included 316 men (32 between 18 and 29, 49 between 30 and 39, 48 between 40 and 49, 48 between 50 and 59, 57 between 60 and 69, 50 between 70 and 79, 32 between 80 and 88 years of age) and 326 women (42 between 18 and 29, 46 between 30 and 39, 57 between 40 and 49, 45 between 50 and 59, 48 between 60 and 69, 58 between 70 and 79, 30 between 80 and 88 years of age).

All participants were cognitively healthy (MMSE > 24) and met the hearing, vision, and English language ability criteria necessary for the completing experimental tasks (Taylor et al., 2017). They were also screened for any neurological and serious psychiatric conditions, as well as for physical conditions or bodily implants that may render their participation unsafe (Taylor et al., 2017). Participants provided informed consent in accordance with the Cambridgeshire Research Ethics Committee (Shafto et al., 2014).

#### 2.1.2 HCP

This sample included 176 unrelated participants, whose data had been released as part of the HCP 1200 subjects data package in March 2017. This sample represented the largest number of participants from the HCP 1200 subjects data release who were unrelated to one another and who had available data on all the demographic, behavioral and fMRI assessments of interest.

The majority of participants (*N* = 163) were right-handed. The sample included 70 younger men (21 between 22 and 25, 35 between 26 and 30, and 14 between 31 and 36 years of age) and 106 younger women (1 between 22 and 25, 49 between 26 and 30, and 56 between 31 and 36 years of age). Although age is presented here in the range format, as advocated by the HCP team (see Van Essen et al., 2012 for the rationale behind this age reporting strategy in HCP data releases), all our brain-behavior analyses used participants’ actual age in years, as available in the HCP restricted data release.

All participants were screened for a history of neurological and psychiatric conditions and use of psychotropic drugs, as well as for physical conditions or bodily implants that may render their participation unsafe. Diagnosis with a mental health disorder and structural abnormalities, as revealed by the MRI structural scans, were also exclusion criteria. Participants provided informed consent in accordance with the HCP research ethics board.

### 2.2 Out-of-Scanner measures

#### 2.2.1 Fluid intelligence

##### 2.2.1.1 Cam-Can

Participants completed the pen-and-paper version of the Cattell Culture Fair Test, Scale 2 Form A (Cattell, 1971; Cattell and Cattell, 1973) in which they had to select the correct answer from multiple alternatives and record it on an answer sheet. The test contains four subtests with distinct non-verbal “puzzles”: series completion, classification, matrices and conditions. Unbeknownst to the participants, each subtest is timed, such that 3, 4, 3 and 2.5 min are allocated for subtest 1, 2, 3 and 4, respectively. Correct responses receive a score of 1 for a maximum score of 46.

##### 2.2.1.2 HCP

Form A of an abbreviated measure of the Raven's Progressive Matrices (RPM; Bilker et al., 2012) gauged participants’ fluid intelligence. This task features patterns made up of 2 × 2, 3 × 3 or 1 × 5 arrangements of squares, with one of the squares missing. Participants must select one of five response choices that best fits the missing square on the pattern. The task has 24 items and 3 bonus items, arranged in order of increasing difficulty. The task is stopped though if the participant makes 5 consecutive incorrect responses. In line with existing guidelines (Bilker et al., 2012; Gray et al., 2003; Gray et al., 2005), total number of correct responses was used as a measure of fluid intelligence.

#### 2.2.2 Crystallized intelligence

##### 2.2.2.1 Cam-Can

The proverb comprehension test was used as an index of crystalized intelligence (Crawford and Stankov, 1996). In this task, participants provide the meaning of three common proverbs in English, which are presented on a computer screen (Shafto et al., 2014). Their responses are recorded digitally and scored by experimenters as incorrect or a “don't know” response (0), partly correct but literal rather than abstract (1), or fully correct and abstract (2). The highest possible score is 6.

#### 2.2.3 Subclinical depression and anxiety

##### 2.2.3.1 HCP

To assess relatively stable subclinical individual differences in depression and anxiety, we used participants' scores on the DSM-oriented depression and anxiety scales (see below for details). Both sets of scores were derived from the participants’ responses to relevant items on the Achenbach Adult Self-Report (ASR) instrument for ages 18–59 (Achenbach, 2009). The ASR contains a total of 123 statements relevant to psychological functioning and requires participants to rate on a 3-point scale (0 *not true*, 1 somewhat or sometimes true, 2 *very true or often true*) how well each item described them over the previous six months. The DSM-oriented depression scale includes items such as "I am unhappy, sad, or depressed”. The DSM-oriented anxiety scale includes items such as " I worry about my future”.

#### 2.2.4 Current negative emotion experience (control measure)

##### 2.2.4.1 HCP

Participants completed the NIH Toolbox Negative Affect Survey, which assesses separately current levels of experienced sadness (e.g., “I felt sad.”, ”I felt like a failure.”), anger (e.g., “I felt angry.”, “I felt bitter about things.”), and fear (e.g., “I felt frightened.’, “I had a racing or pounding heart.”), respectively. The measure requires participants to rate on a 5-point scale (1 *never* to 5 *always*) how often they experienced the relevant emotion within the past seven days. Scores on the sadness, anger and fear subscales were averaged to create an index of current negative emotional state, which was entered as a covariate in the relevant hypothesis testing analysis (see Section 2.7.1.2 for details).

### 2.3 In-Scanner task

#### 2.3.1 Cam-Can

Participants watched a shortened version of Alfred Hitchcock's black-and-white television drama “Bang! You're Dead” (Hitchcock, 1961; Hasson et al., 2008, 2010) edited from 30 min to 8 min while maintaining the plot (Shafto et al., 2014). The movie was selected to be engaging and unfamiliar to the participants.

#### 2.3.2 HCP

Participants completed four movie viewing runs over two scanning sessions on two separate days. Each run was about 15 min long and comprised 1 to 4.3 min long excerpts from Hollywood movies, as prepared and published by Cutting et al. (2012), and independent films, freely available under Creative Commons Licensing. All four movie runs include a Vimeo repeat clip at the end, which was not included in the analyses. In each run, 20 s of rest (i.e., black screen with “REST” in white text) precede the beginning and follow the end of each movie clip.

##### 2.3.2.1 HCP movie features

To quantify concrete featural fluctuations in the movie task, we used the output of the semantic feature coding conducted by Jack Gallant's laboratory (cf. Huth et al., 2012) and available as part of the HCP1200 Subjects Data Release. By using this semantic feature coding, we were able to characterize the extent to which window-to-window FC reconfiguration is yoked to ongoing, meaningful fluctuations in the external environment. Each movie frame is coded for the presence/absence (0/1) of 859 semantic elements recorded as nouns (*N* = 629), verbs (*N* = 229) and adjective(s) (*N* = 1). Our analyses focused on semantic features listed as nouns, which we took to reflect environmental attributes (e.g., presence of objects, people, buildings) and semantic features listed as verbs, which reflected actions performed by the movie characters or natural phenomena. As we detail below, the brain data corresponding to each movie was broken down into 40 s second sliding windows, moved in increments of 2 s. The binary semantic feature matrices corresponding to each sliding window from the brain data were averaged. In these averaged matrices, the value in each cell signified the percentage of frames within a sliding window when an entitity or action was present. As in the brain data, window-to-window similarity in semantic features was indexed with the adjusted mutual information index (AMI) (Vinh et al., 2012), with each unique value in the averaged semantic features for each window acting as a community/cluster label.

### 2.4 fMRI data acquisition

#### 2.4.1 Cam-Can

Images were acquired with a 3T Siemens TIM Trio System (32-channel coil). T1-weighted anatomical scans were acquired with a MPRAGE sequence (TR = 2250 ms, TE = 2.99 ms, flip angle=9°, FOV = 256 mm x 240 mm x 192 mm, 1 mm isotropic voxels, GRAPPA acceleration factor = 2). Functional images were acquired with a multi-echo EPI sequence (TR=2470 ms, [TE=9.4 ms, 21.2 ms, 33 ms, 45 ms, 57 ms], flip angle=78°, FOV = 192 × 192 mm, 32 axial slices of 3.7 mm thickness, acquired in descending order with 20% gap, voxel size of 3 mm × 3 mm x 4.4 mm).

#### 2.4.2 HCP

Images were acquired with a Siemens 7T scanner housed at Washington University in St. Louis (32-channel coil). Pulse and respiration were measured during scanning. Functional images were acquired with a multiband EPI sequence (TR=1000 ms, TE=22.2 ms, flip angle=45°, FOV = 208 × 208 mm, 85 slices of 1.6 × 1.6 mm in-plane resolution, 1.6 mm thick, no gap, multiband acceleration factor of 5). Two of the movie runs were acquired with an anterior-to-posterior, while the other two with a posterior-to-anterior, phase encoding sequence (so that phase encoding sequence effects could cancel each other out over all the runs).

### 2.5 fMRI data preprocessing

**Fig. 1:**
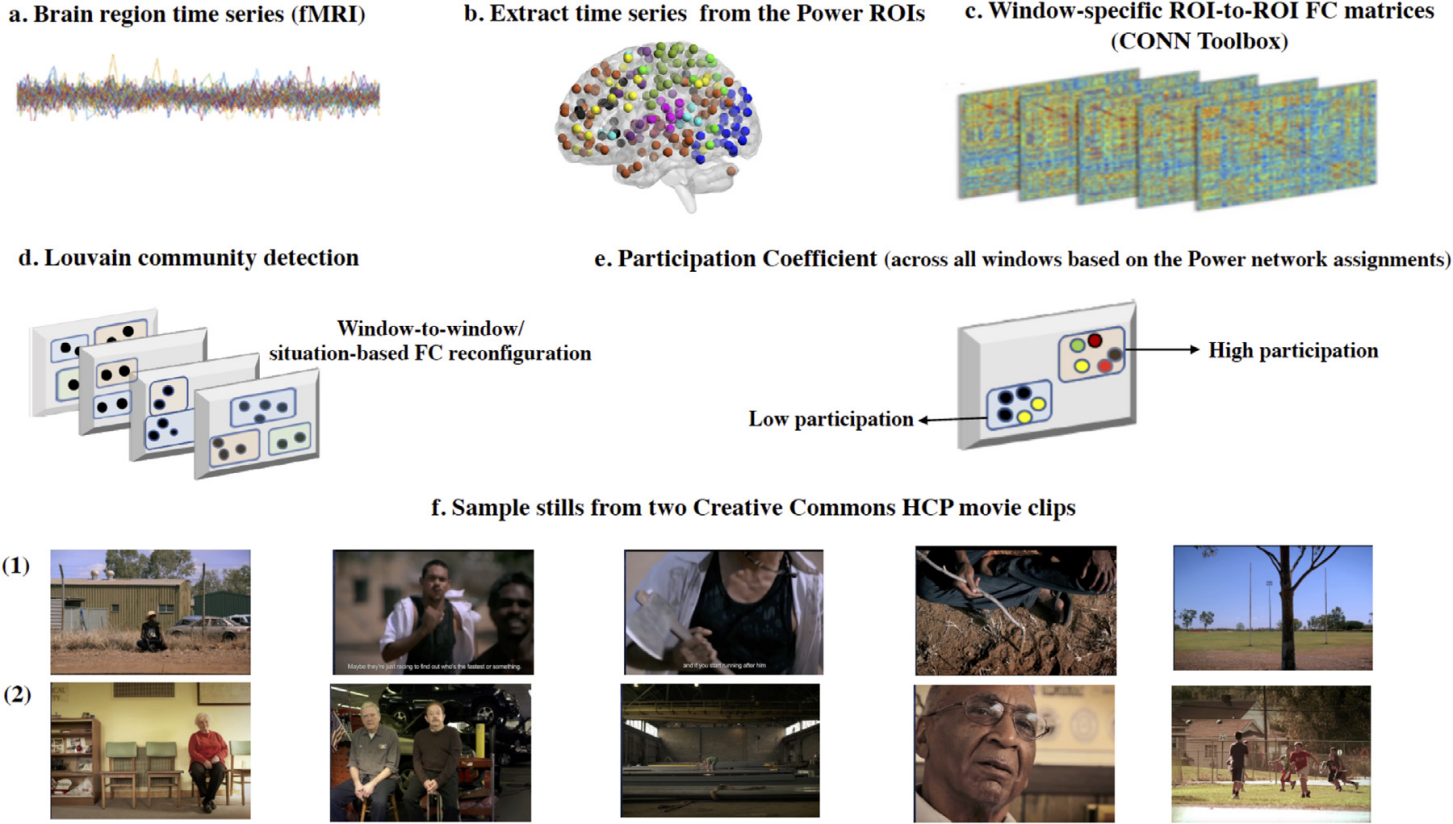
. Schematic representation of the main preprocessing and analysis steps across both samples (panels a-e). HCP CC movie stills (panel f)*.*

The main preprocessing and analysis steps for both samples are presented in Fig. 1.

#### 2.5.1 Initial preprocessing

##### 2.5.1.1 Cam-Can

Preprocessing began with the averaging of the corresponding images from the multiple echos. We opted for a simple average, rather than a weighted sum, in order to maximize comparability with the HCP data, which did not contain multiple echos. We reasoned that our strategy was defensible in light of evidence that simple averaging yields similar improvements in 3T image quality as weighted summing (Kettinger et al., 2016; Poser et al., 2006). Subsequently, we performed image processing in SPM12 (Wellcome Department of Imaging Neuroscience, London, UK). Specifically, we corrected for slice timing differences and rigid body motion (which included unwarping), spatially normalized the images to the standard Montreal Neurological Institute (MNI)−152 template, and smoothed them (full-width half-maximum, 6 mm).

##### 2.5.1.2 HCP

The present report used the minimally preprocessed movie watching data from the HCP 1200 subjects data release. These data have been preprocessed with version 3 of the HCP spatial and temporal pipelines (Smith et al., 2013; for specification of preprocessing pipeline version, see http://www.humanconnectome.org/data). Spatial preprocessing involved removal of spatial and gradient distortions, correction for participant movement, bias field removal, spatial normalization to the standard Montreal Neurological Institute (MNI)−152 template (2 mm isotropic voxels), intensity normalization to a global mean and masking out of non-brain voxels. Subsequent temporal preprocessing steps involved weak high-pass temporal filtering with the goal of removing linear trends in the data.

#### 2.5.2 Additional denoising

Because motion can significantly impact FC measures (Power et al., 2012; Van Dijk et al., 2012), we implemented several additional preprocessing steps to address this potential confound in both samples. First, after extracting the BOLD time series from our regions-of-interest (ROIs, see below), but prior to computing the ROI-to-ROI correlations, we used the Denoising step in the CONN toolbox (version 17c; Whitfield-Gabrieli and Nieto-Castanon, 2012) to apply further physiological and rigid motion corrections. Specifically, linear regression was used to remove from the BOLD time series of each ROI the BOLD time series of the voxels within the MNI-152 white matter and CSF masks, respectively (i.e., the default CONN option of five CompCor-extracted principal components for each [ten principal components in total], Behzadi et al., 2007), the 6 realignment parameters, their first-order temporal derivatives and their associated quadratic terms (24 regressors in total, cf. Bolt et al., 2017). The residual BOLD time series were bandpass filtered (0.008 Hz< *f* < 0.09 Hz), linearly detrended and despiked by applying a continuous “squashing” (i.e., hyperbolic tangent) function to all the data points (all three are default CONN denoising steps, Whietfield-Gabrieli and Castanon, 2012). Following these corrections (which did not include global signal regression) (Murphy and Fox, 2017), an inspection of each subject's histogram of voxel-to-voxel connectivity values revealed a normal distribution, approximately centered around zero, which would suggest reduced contamination from physiological and motion-related confounds (cf. Whietfield-Gabrieli and Castanon, 2012). Nonetheless, in all hypothesis testing analyses, we controlled for the average relative (i.e., volume-to-volume) displacement per participant, a motion metric widely recommended for use in order to minimize any potential residual influence of motion on the effects of interest (Power et al., 2012, 2015; Satterthwaite et al., 2013).

##### 2.5.2.1 HCP-unique

Unlike the Cam-Can, the HCP dataset contained multiple movie clips in the same run. Consequently, in order to isolate movie-related functional coupling from mere co-activation effects corresponding to the beginning and end of a movie clip (i.e., two regions that are both activated at the beginning of a movie clip and de-activated at its end, although they do not “communicate” with one another throughout the movie clip), we created an additional regressor for the HCP ROIs. This regressor reflected the main movie effects and was obtained by convolving a boxcar task design function with the hemodynamic response function, and their first temporal order derivative (cf. Braun et al., 2015; Vatansever et al., 2015; Westphal, Wang, & Rissman, 2017).

### 2.6 fMRI data analysis

#### 2.6.1 ROI definition

229 nodes for 10 core large-scale functional brain networks (i.e., default-mode [DMN], frontoparietal [FPC], cingulo-opercular [CON], salience [SAL], dorsal attention [DAN], ventral attention [VAN], somatomotor [SM], subcortical [SUB], auditory [AUD] and visual [VIS]) were defined for each participant as spherical ROIs (radius 5 mm) centered on the coordinates of the regions reported in Power et al. (2011) and assigned network labels corresponding to the graph analyses from this earlier article. We selected the Power et al. atlas because it was created by taking into account both the task-related activation (derived meta-analytically) and the resting state connectivity patterns of the component voxels for each ROI. Thus, this atlas provided an optimal parcellation scheme for charactering FC reconfiguration during a naturalistic cognition condition that, due to the lack of an explicit task, was likely to share significant similarities with a resting state condition (Vanderwal et al., 2017).

The ROIs were created in FSL (Smith et al., 2004), using its standard 2 mm isotropic space, with each ROI containing 81 voxels. These template space dimensions were selected because they yielded the most adequate spatial representation of the Power atlas. The 229 ROIs represent a subset of the 264 putative functional areas proposed by Power et al. (2011). The 229 ROIs were selected because, based on Power et al.’s analyses, they showed relatively unambiguous membership to one of the ten large-scale functional networks outlined above.

##### 2.6.1.1 Cam-CAN

Because the Power atlas was validated in young adult samples, we tested the fit of its ROIs and of its network assignments to the Cam-CAN lifespan data prior to running all the analyses of interest (see “Fit of the Power atlas to the Cam-CAN dataset”, “Fit of the Power network assignment to the Cam-CAN dataset” in the Supplementary Materials). These analyses demonstrated the good fit of the Power ROIs and of its network assignments to the Cam-CAN lifespan dataset. However, there was evidence of a modest, but significant negative relationship between age and the average functional homogeneity of the Power ROIs (Spearman's *rho* of −0.21, *p* = 10^−5^). Consequently, an individual-specific summary measure of ROI functional homogeneity was introduced as a covariate in all hypothesis testing analyses.

#### 2.6.2 ROI-to-ROI correlations in timeseries: CONN toolbox

Pairwise coupling among the 229 ROIs was estimated in CONN and expressed as Fisher's z-transfomed correlation coefficients. Following existing practices aimed at maximizing interpretability of results in network neuroscience studies of lifespan differences in functional brain architecture (Betzel et al., 2014), we did not threshold the ROI-to-ROI correlation coefficients in order to ensure that the same connections and, thus, the same statistical power would be available across all participants. Instead, we used both positive and negative Fisher's z-transformed correlation coefficients to compute the indices of interest for all connectivity analyses. We reasoned that such an approach would be particularly well-justified in our present case since global signal regression, an artefact removal technique that generates negative correlations whose interpretation is still controversial, was not part of our preprocessing pipeline (for further discussion on the validity of the negative correlations obtained with the CONN toolbox, see Whitfield-Gabrieli and Nieto-Castanon, 2012).

To characterize individual differences in dynamic FC-based community structure, we used a combined sliding window and clustering based approach in both samples (Iraji et al., 2020; Lurie et al., 2020). Thus, pairwise coupling among the 229 ROIs was estimated in CONN using a sliding window of 40 s in length, with approximately 2 s gap in-between windows (2.47 s in the Cam-CAN, 2 s in the HCP) and a "hanning weighting" (i.e., greater weight to the scans in the middle of the window relative to the ones at the periphery) applied to all the time points within a window. The use of a hanning weighting was intended to reduce the autocorrelation in the fMRI data series, thereby maximizing the opportunity to detect differences in FC-based community structure between adjacent windows.

The window length was selected with the goal of augmenting sensitivity to patterns of dynamic functional brain reconfiguration and individual differences in such processes. Prior research suggested that sliding windows around 30 s long would meet both criteria (e.g., Braun et al., 2015; J. Chen et al., 2016; Leonardi & Van De Ville, 2015; Preti et al., 2017; Telesford et al., 2016). However, to enable cross-sample comparisons, the same window length was required in both datasets. In the Cam-CAN, a 30-s window meant that ROI-to-ROI correlations in each window would have been based on a number of data points (i.e., 12 vol) which was insufficient for obtaining robust metrics. Consequently, we opted for a 40-s window as an acceptable compromise between sensitivity to individual differences in dynamic functional brain reconfiguration and statistical power to characterize robust ROI-to-ROI correlations in each sliding window for both datasets.

##### 2.6.2.1 Cam-CAN

The movie was broken down into 177 partially overlapping windows.

##### 2.6.2.1 HCP

Periods of rest between consecutive movie clips were eliminated from the analyses. Each of the 14 movie clips was separately broken down into partially overlapping windows for a total of 1152 windows across all 14 movies.

#### 2.6.3 Network-level analyses

All the network-level metrics were computed using the Brain Connectivity Toolbox (BCT, Rubinov and Sporns, 2010) and the Network Community Toolbox (Bassett, D.S. [2017, November]. Network Community Toolbox. Retrieved from http://commdetect.weebly.com/), as described below.

##### 2.6.3.1 Functional community structure: Louvain algorithm

The optimal whole-brain division into non-overlapping communities was estimated using a Louvain community detection algorithm implemented in the BCT (Rubinov and Sporns, 2010). This algorithm partitions a network into non-overlapping groups of nodes with the goal of maximizing an objective modularity quality function, Q (Betzel and Bassett, 2017; Rubinov and Sporns, 2011; Sporns and Betzel, 2016). Following prior proposals regarding the greater biological significance of positive ROI-to-ROI connections, we implemented the Louvain algorithm by using the adapted modularity function Q*, proposed by Rubinov and Sporns (2011), which has since been widely used (e.g., J. Chen et al., 2016; Fukushima and Sporns, 2020; Tooley et al., 2020), including in studies of lifespan differences in functional brain architecture (Betzel et al., 2014). In this formulation, the contribution of positive weights to Q is not affected by the presence of negative weights in the network, whereas the contribution of negative weights to Q decreases with an increase in positive weights (for further details on the procedure, see “Community detection” in the Supplementary Materials). Thus, this modularity estimation procedure retains the focus on positive weights, which are regarded as holding greater biological significance, similar to thresholding-based approaches which eliminate negative weights, however, it circumvents the problem of unequal statististical power (i.e., unequal number of connections contributing to model specification) across participants (cf. Betzel et al., 2014). To minimize potential confounds associated with the estimation of additional free parameters (e.g., the temporal coupling parameter between two adjacent temporal windows in multilayer community detection algorithms, Bassett et al., 2011; Braun et al., 2015; Mucha et al., 2010), we used the single-layer procedure to estimate community structure independently in each sliding window (see also J. Chen et al., 2016).

To account for the near degeneracy of the modularity landscape (Good et al., 2010) and for changes in community structure due to variations in the estimation parameters, the community detection algorithm was initiated 100 times for each of the three values of the spatial resolution parameter, centered around the default value of 1 (i.e., 0.95, 1.00, 1.05, cf. Betzel and Bassett, 2017; Braun et al., 2015; J. Chen et al., 2016). Based on these analyses, run separately for each of the three spatial resolution values, an agreement matrix, denoting the probability of each ROI pair to belong to the same functional community, was computed for each participant in each movie window. Subsequently, a consensus partition (i.e., whole-brain division into constituent communities) was estimated for each participant in each movie window by applying a Louvain community detection algoritm (100 repetitions and a threshold tau of 0) to the corresponding agreement matrix (cf. Bassett et al., 2013; Lancichinetti & Fortunato, 2012).

##### 2.6.3.2 FC variability: window-to-window

Using the Network Community Toolbox, we estimated similarity in FC-based community structure (derived with the Louvain algorithm, as described above) between consecutive windows using the adjusted normalized mutual information index [AMI], corrected for chance (Vinh et al., 2010). An index of window-to-window FC reconfiguration was computed by subtracting from 1 the average AMI across all pairs of temporally adjacent windows. This index combines spontaneous (i.e., stimulus-independent) window-to-window reconfiguration with reconfiguration driven by window-to-window concrete featural fluctuations (e.g., presence/absence of objects, people).

##### 2.6.3.3 FC variability: event boundary-based (Cam-CAN only)

Employing the event boundaries identified by independent raters in Ben-Yakov and Henson (2018) with a keypress when they felt that “one event [meaningful unit] ended and another began”, we selected pairs of non-overlapping 40 s windows, separated by ~ 5 to 7 s, which belonged to adjacent narrative segments (12 windows in total). FC reconfiguration in response to these high-level event boundaries was estimated by subtracting from 1 the average AMI across all such pairs of temporally adjacent windows. This index reflects FC reconfiguration related to event boundaries, as well as stimulus-independent and lower-level reconfiguration indicative of featural changes (e.g., presence/absence of objects, people).

Due to the shorter duration and the structure of the HCP movie clips, narrative event boundaries were less legible and, as such, event boundary-based FC reconfiguration did not constitute a point of inquiry in the HCP data.

##### 2.6.3.4 Functional network interactions: participation coefficient

To characterize patterns of functional network interaction linked to FC variability, we used the participant coefficient. The participation coefficient assesses the diversity of a node's intermodular connections (i.e., the extent to which a node interacts with nodes outside its native community) (J. Chen et al., 2016; Rubinov and Sporns, 2010). Here, a node's native community was the one to which it was assigned in Power et al. (2011), the study that validated the functional atlas. A node's participation coefficient was based on the consensus partitions corresponding to each of the 177 (Cam-CAN) or 1152 (HCP) sliding windows and was given by the formula

$$P_{i}=1-\sum_{m}\in M{\left(\frac{k_{i}\left(m\right)}{k_{i}}\right)}^{2},\;\;\text{where}$$

P_i_ is the participation coefficient of node i, M is the set of communities from a given partition (in our case, the whole-brain partition into communities, as described by Power et al., [2011]), k_i_(m) corresponds to the number of times that node i and all the nodes in community m have been assigned to the same community across all time windows, and k_i_ is the number of times that node i and the remaining 228 nodes have been assigned to the same community across all time windows. In our case, higher participation coefficients characterised nodes that tended to show a roughly equal number of interactions (where interaction means assignment to the same community within a sliding window) with nodes from all the functional networks identified by Power et al. In other words, in each sliding window, higher participation nodes underpin the communication among the different functional networks characterised by Power et al.

##### 2.6.3.5 Coupling between window-to-window FC and concrete environmental variability (HCP only)

To index coupling between FC and concrete environmental variability, we computed the Spearman's rank correlation between window-to-window similarity in FC-based community structure and window-to-window noun-/verb-based semantic similarity. Because we were specifically interested in brain-(noun/verb-based) movie *coupling*, overall window-to-window FC reconfiguration was regressed out from both indices. The two residual brain-movie (noun- vs. verb-based) couplings were used in all the reported analyses.

###### 2.6.3.5.1 Reliability analyses (HCP only)

To test whether a unitary construct can be extracted for each neural index of interest across all 14 movies, we conducted separate reliability analyses on the 42 values associated with each index (i.e., three values for each of the 14 movies, corresponding to the community detection estimates obtained with a spatial resolution parameter of 0.95, 1, 1.05). Since subject motion can impact such reliability estimates, we present the relevant Cronbach's alpha values, both before and after regressing out subject level average frame-to-frame displacement (see Preprocessing above for the additional motion effect removal procedures already implemented). Additionally, to reflect the variables used in our analyses, for the brain- (noun/verb-based) movie coupling, we present reliability estimates based on data from which we regressed out both subject motion and spontaneous window-to-window FC reconfiguration.

For the window-to-window FC similarity index, we obtained Cronbach's alphas of 0.87 and of 0.89 (with regression of the motion summary statistic). The brain-(noun) movie coupling index showed Cronbach's alphas of 0.66 and of 0.68 (with regression of motion and window-to-window brain reconfiguration). For the brain-(verb) movie coupling index, we observed Cronbach's alphas of 0.65 and of 0.62 (with regression of motion and window-to-window brain reconfiguration). Across all 229 ROIs, the participation coefficients showed Cronbach's alphas between 0.76 and 0.98 (both with and without regression of the summary motion statistic).

### 2.7 Brain-behavior analyses

#### 2.7.1 Canonical correlation analysis (CCA)

To characterize the relationships among FC reconfiguration (i.e., changes in community structure), network-level diversity in functional interactions, age, cognition and affect (fluid intelligence, depression, anxiety), we conducted a series of canonical correlation analyses (CCAs, Hotelling, 1936) with cross-validation procedures (cf. Hair et al., 2009). CCA is a multivariate technique, which seeks maximal correlations between two sets of variables by creating linear combinations (i.e., canonical variates) from the variables within each set. Recently, CCA has been successfully used to investigate brain-behavior relationships in large datasets (see Smith et al., 2015; Tsvetanov et al., 2016; Wang et al., 2020). CCA was implemented in Matlab using the canoncorr module. In order to obtain reliable estimates of correlations between the brain or behavioral variables and their corresponding variates, it is generally recommended that CCA be performed on a sample size at least ten times the number of variables in the analysis (Hair et al., 1998), a criterion which was exceeded in all analyses reported below.

The performance of our CCA-derived models was tested by using a 10-fold cross validation procedure. For all sets of CCAs, discovery analyses were conducted on nine folds of data and the resulting CCA weights were employed to derive predicted values of the brain and behavioral variate in the left-out (“test”) fold. This procedure was repeated until each of the ten folds served as “test” data once. The correlation between the predicted brain and behavioral variates across all testing folds was evaluated using a permutation test with 100,000 samples (cf. Smith et al., 2015). To describe the relationship between the behavioral or brain variables and their corresponding variates across all the testing folds, we include correlations between the observed value of a brain or behavioral variable and the predicted value of its corresponding variate, as well as standardized coefficients, analogous to multiple regression coefficients, which indicate the unique association between the observed value of a behavioral or brain variable and the predicted value of its corresponding variate. 95% confidence intervals (CI) for each correlation and standardized regression-like coefficient were obtained by using the bootci function in Matlab (with default settings and 100,000 bootstrap samples). The correlation and standardized regression-like coefficients described above are analogous to canonical loadings and canonical weights, respectively (see also Tsvetanov et al., 2016; D. Vatansever et al., 2017), with the only difference being that they are computed in the test, rather than the discovery, folds and, thus, reflect more conservative effect estimates.

##### 2.7.1.1 Cam-CAN

In the CCAs involving age, network participation and FC reconfiguration, the data were broken down into ten folds, all but two containing 64 participants for a total of 642 participants. For the CCAs involving fluid and crystallised intelligence, all but three of the ten folds of testing data contained 61 participants for a total of 613 participants. For all CCA sets, when evaluating the relationship between the predicted values of the two variates or the relationship between the observed value of each variable and the predicted value of its corresponding variate, we controlled for gender, handedness, subject-specific motion and subject-specific ROI functional homogeneity (RSC).

##### 2.7.1.2 HCP

The data were broken down into ten folds, six of which contained 18 participants (the remaining folds contained 17 participants each). In all sets of CCAs, when evaluating the relationship between the predicted values of the two variates or the relationship between the observed value of each variable and the predicted value of its corresponding variate, we controlled for gender, handedness, subject-specific motion, and years of education. In CCA 3 (see Fig. 6) we additionally controlled for current negative emotional experience to ensure that the observed associations are not due to global negative mood around the time of testing.

#### 2.7.2 Partial least squares analysis (PLS; Cam-CAN only)

To identify patterns of ROI participation that are specific to FC reconfiguration related to concrete featural versus more abstract event boundaries, we used partial least squares correlation often referred to as *PLS* (Krishnan et al., 2011), a multivariate technique that can identify in an unconstrained, data-driven manner, neural patterns (i.e., latent variables or LVs) related to individual differences variables (behavioral PLS). PLS was implemented using a series of Matlab scripts, which are available for download at https://www.rotman-baycrest.on.ca/index.php?section=345. In the behavioral PLS analyses we conducted, one matrix comprised residual scores on the event boundary-based FC reconfiguration index (i.e., average [1- AMI] across all neighbouring windows from different narrative segments from which age and average window-to-window FC reconfiguration were partialled out) (PLS 1) or average window-to-window functional brain reorganization (i.e., average [1- AMI] across all temporally adjacent windows from which age and average functional brain reorganization linked to event boundaries were partialled out) (PLS 2, presented in the Supplemental Materials), whereas the second matrix contained each participant's ROI participation matrices (Krishnan et al., 2011). Each matrix entry corresponded to the participation coefficient of one ROI from one subject.

In all the reported analyses, the significance of each LV was determined using a permutation test with 100,000 permutations (in the permutation test, the rows of the ROI participation data are randomly reordered, Krishnan et al., 2011). In the case of our present analyses, PLS assigned to each ROI a weight, which reflected the respective ROI's contribution to a specific LV. The reliability of each ROI's contribution to a particular LV was tested by submitting all weights to a bootstrap estimation (100,000 bootstraps) of the standard errors (SEs, Efron, 1981) (the bootstrap samples were obtained by sampling with replacement from the participants, Krishnan et al., 2011). We opted to use 100,000 permutations and 100,000 bootstrap samples (the same value used for the other bootstrapping and permutation-based testing herein reported) in order to increase the stability of the reported results, since these parameters are several orders greater than the standard ones (i.e., 500 permutations/100 bootstrap samples), recommended by McIntosh and Lobaugh (2004) for use in PLS analyses of neuroimaging data. A bootstrap ratio (BSR) (weight/SE) of at least 3 in absolute value was used as a threshold for identifying those ROIs that made a significant contribution to the identified LVs. The BSR is analogous to a z-score, so an absolute value greater than 2 is thought to make a reliable contribution to the LV (Krishnan et al., 2011), although for neuroimaging data BSR absolute values greater than 3 tend to be used (McIntosh & Lobaugh, 2004).

Below we present the effects obtained with the aforementioned canonical procedures for establishing validity and reliability of the PLS results. However, in the Supplemental Materials, we further document the robustness of our findings by demonstrating that the identified brain LVs are replicated when we apply to the PLS analyses a 10-fold cross-validation procedure similar to the one employed with the CCAs.

## 3 Results

### 3.1 Fluid intelligence and FC variability across adulthood

**Fig. 2:**
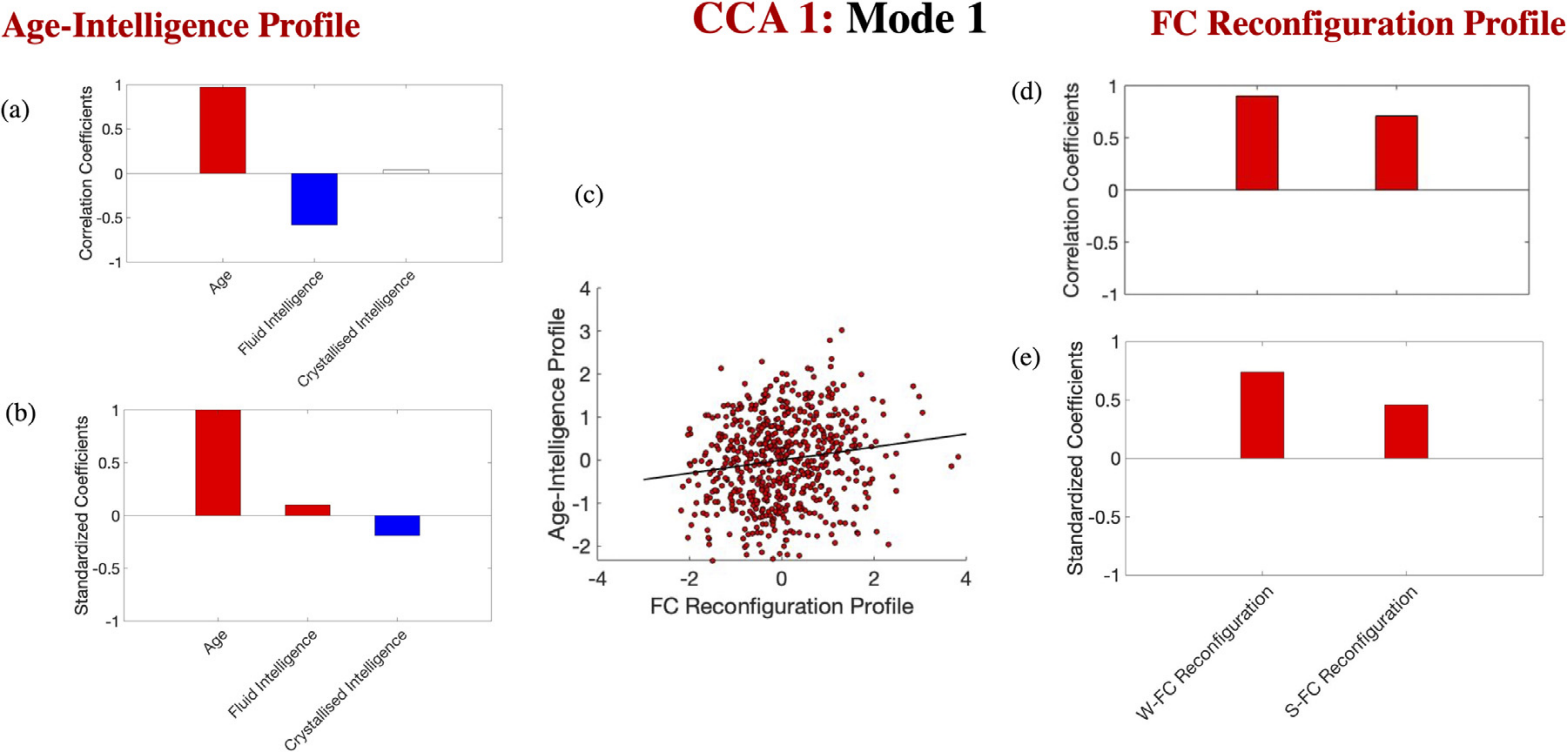
*Lifespan differences in FC reconfiguration and intelligence.* Correlation [panels a, d] and standardised coefficients [panels b, e] describing the relationship between the observed variables and the predicted value of their corresponding canonical variate across all test CCAs. The scatter plot in panel (c) describes the linear relationship between the predicted values of the two variates across all test CCAs and is based on standardised variables. In panel (a), white boxes correspond to coefficients that were not robust across all the bootstrapping samples. Gender, handedness, ROI homogeneity and the summary motion metric were introduced as covariates. W-FC Reconfiguration= window-to-window FC reconfiguration. S-FC = situation-based FC reconfiguration.

***Window-to-window and event boundary-based FC variability increase across the adult lifespan; neither is linked to fluid/crystallised intelligence beyond age (Cam-CAN).*** We ran ten discovery CCAs to characterize the relationship of window-to-window and event boundary-based FC reconfiguration with fluid intelligence, as well as age and crystallised intelligence. The discovery CCAs identified one significant mode, which was validated across all test sets (*r* of 0.15, *p* = 7 * 10^−5^). This mode indicated that greater FC reconfiguration (i.e., reduced similarity in community structure between temporally adjacent windows, as well as neighbouring windows from distinct narrative segments) typifies older individuals with lower fluid intelligence scores (see Fig. 2-a, d). An inspection of the standardized coefficients revealed that the link between greater FC variability and fluid ability is mostly due to age (i.e., older individuals tend to have lower fluid intelligence scores relative to younger individuals, see Fig. 2-b, e).

*Fluid intelligence is not associated with window-to-window FC reconfiguration: Confirmation in the HCP.* A partial correlation analysis conducted on the HCP data, in which we controlled for gender, handedness, education, age and the summary motion metric, confirmed the lack of statistically significant association between window-to-window FC reconfiguration and fluid intelligence, *r*(174) = 0.10, permutation-based *p* > .18.

### 3.2 Fluid intelligence and brain network interactions underpinning FC variability across adulthood

**Fig. 3:**
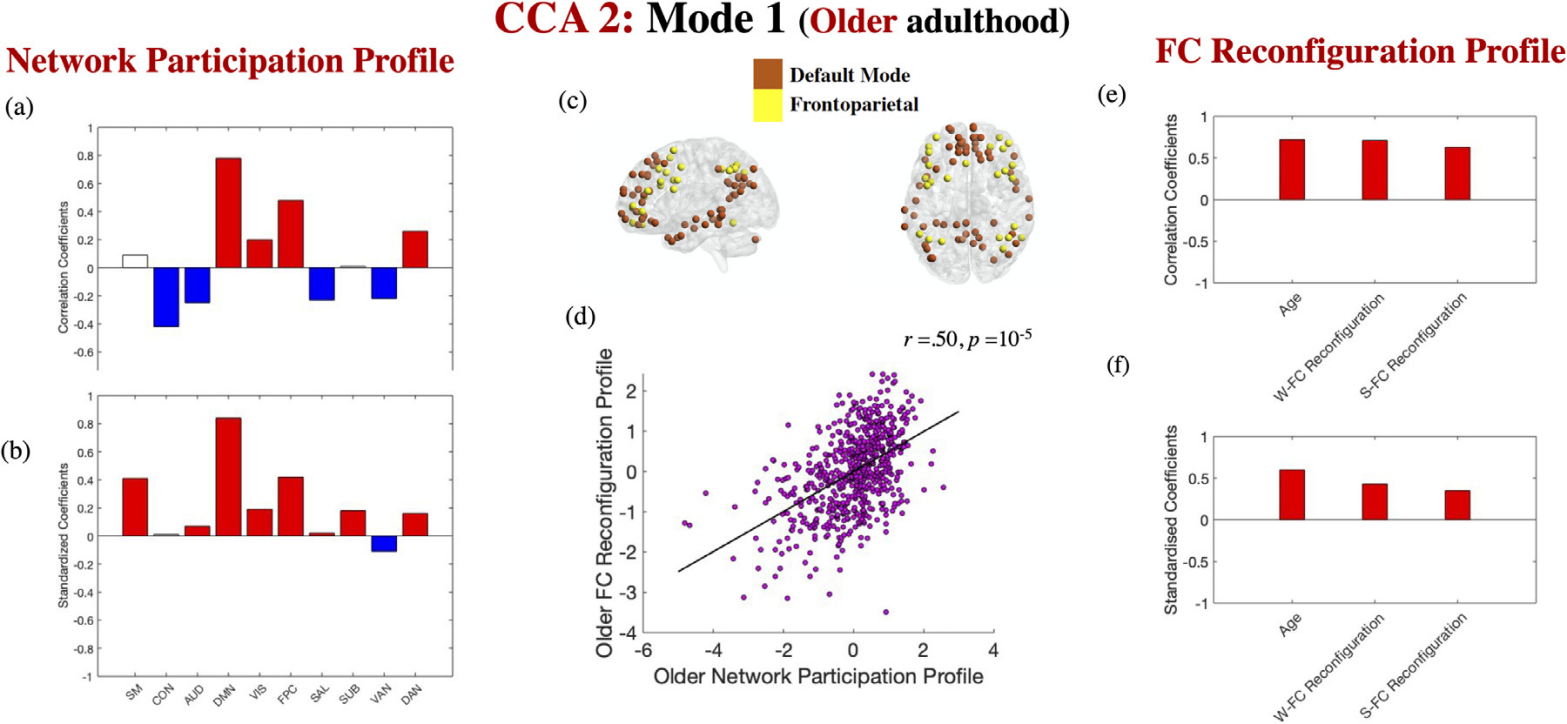
*FC reconfiguration and network participation in older adulthood*. Correlation [panels a, e] and standardised coefficients [panels b, f] describing the relationship between the observed variables and the predicted value of their corresponding canonical variate across all test CCAs. Panel (c) depicts the two networks from the Power atlas showing the highest correlation with the brain variate in panel (a). The scatter plot in panel (d) describes the linear relationship between the predicted values of the two variates across all test CCAs and is based on standardised variables. In panels (a) and (b), white boxes correspond to coefficients that were not robust across all the bootstrapping samples. Gender, handedness, ROI homogeneity and the summary motion metric were introduced as covariates. W-FC Reconfiguration= window-to-window FC reconfiguration. S-FC = situation-based FC reconfiguration.

**Fig. 4:**
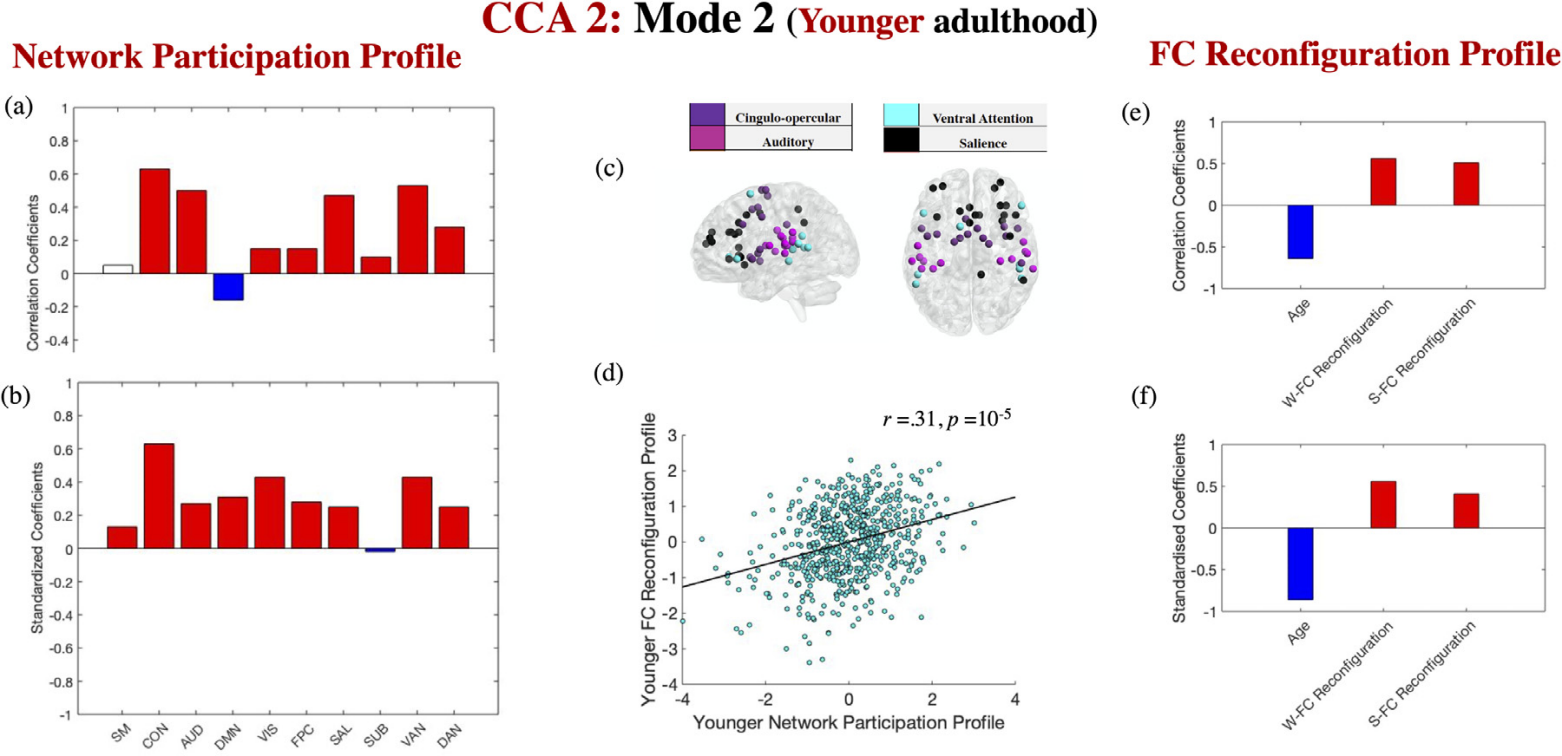
*FC reconfiguration and network participation in younger adulthood.* Correlation [panels a, e] and standardised coefficients [panels b, f] describing the relationship between the observed variables and the predicted value of their corresponding canonical variate across all test CCAs. Panel (c) depicts the four networks from the Power atlas showing the highest correlation with the brain variate in panel (a).The scatter plot in panel (d) describes the linear relationship between the predicted values of the two variates across all test CCAs and is based on standardised variables. In panel (a), white boxes correspond to coefficients that were not robust across all the bootstrapping samples. Gender, handedness, ROI homogeneity and the summary motion metric were introduced as covariates. W-FC Reconfiguration= window-to-window FC reconfiguration. S-FC = situation-based FC reconfiguration.

***Fluid (but not crystallised) intelligence is linked to the profile of functional network interaction underlying both types of FC variability in young adulthood; this relationship is independent of age and mean FC variability (Cam-CAN).*** Ten discovery CCAs were conducted to probe the relationship between diversity in the functional interactions of the ten networks from the Power atlas (i.e., the average participation coefficient across all the ROIs within each network) and age-linked patterns of window-to-window versus event boundary-based FC reconfiguration. The discovery CCAs detected two significant modes, which were validated across all test sets (*r*s of 0.50 and 0.31, respectively, both *p*s of 10^−5^). The first mode indicated that, at older ages, greater FC reconfiguration is associated with greater participation of networks involved in self-guided cognition and creation of situational models during event perception (DMN), as well as top-down control (FPC) and attention (DAN), but reduced participation of networks implicated in environmentally driven processing (CON, AUD, SAL and VAN, see Fig. 3). The second mode suggested that, at younger ages, stronger FC reconfiguration was linked to greater global participation, but particularly for the network involved in environmental vigilance and control maintenance (i.e., CON, cf. Fig. 4).

**Fig. 5:**
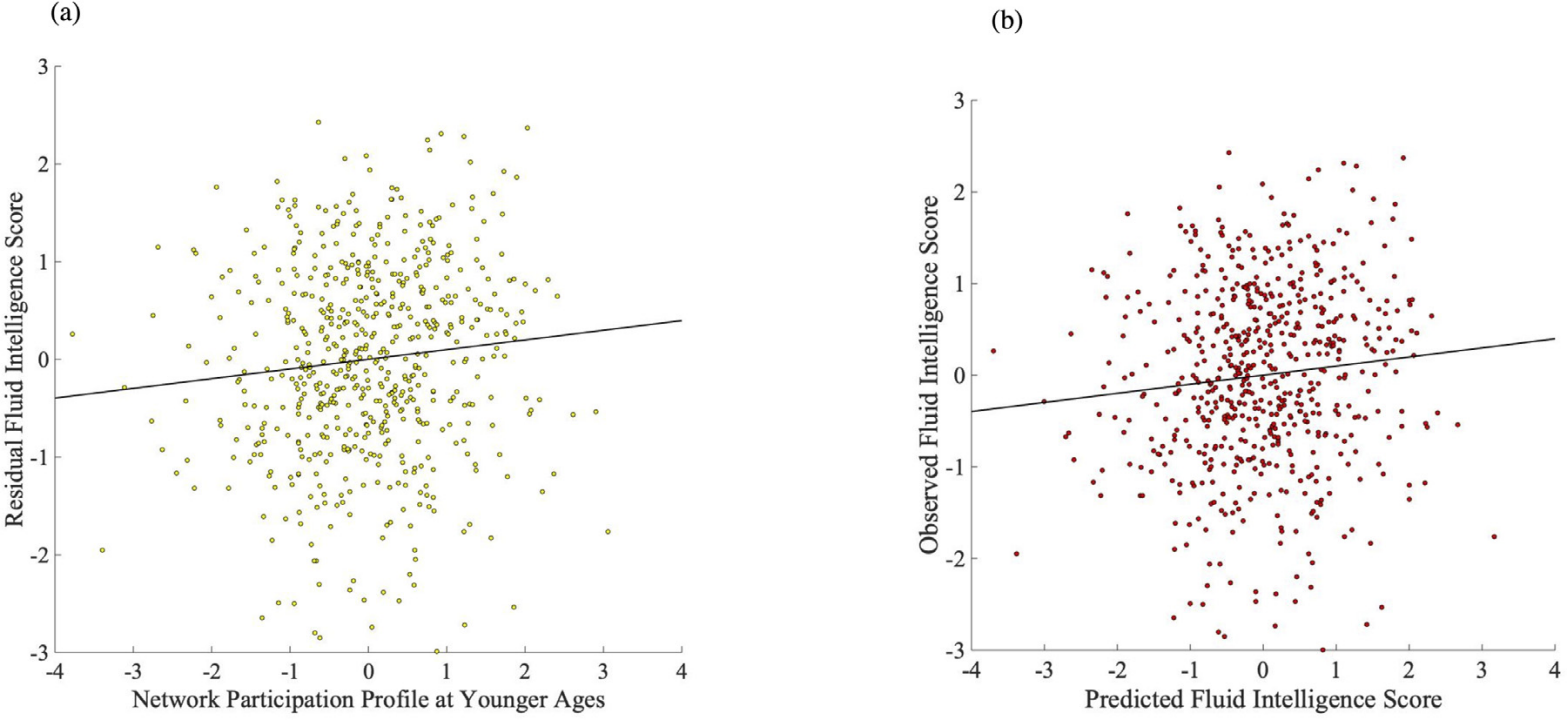
Scatterplot describing the association between fluid intelligence and the network participation profile linked to FC reconfiguration in younger adulthood, after controlling for age, gender, handedness, ROI functional homogeneity (RSC), summary motion statistic, crystallised intelligence, window-to-window and situation-based FC reconfiguration, as well as the network participation profile linked to FC reconfiguration in older adulthood. Panel (a) depicts the results of the original regression analysis, whereas panel (b) presents the results of the cross-validated regression analyses.

A robust regression analysis, conducted in Matlab with default settings (bisquare robust fitting weight function with a tuning constant of 4.685) and using fluid intelligence as the outcome, revealed its significant positive association with the network participation profile linked to FC reconfiguration during younger, (*b* = 0.083, *SE* = 0.031, *t*(602) = 2.643, *p* = 0.008), but not older (*b* = −0.053, *SE* = 0.037, *t*(602) = −1.433, *p* = 0.153), ages (covariates included window-to-window and event boundary-based FC reconfiguration, respectively, age, sex, handedness, crystallised intelligence, the summary motion metric and the summary ROI homogeneity metric) (see Fig. 5-a). The corresponding robust regression analysis using crystallised intelligence as the outcome unveiled no significant associations with either participation profile (both *p*s > 0.66). In neither robust regression analysis did levels of window-to-window or event boundary-based FC reconfiguration made a significant contribution to either fluid or crystallised intelligence (all *p*s > 0.25).

To confirm the association between fluid intelligence and the young adult participation profile, we conducted a robust regression on only these two variables using the same ten-fold cross-validation procedure implemented in the CCAs. Specifically, the regression coefficient of fluid intelligence on the young participation profile was estimated on nine folds of data (the left-out tenth fold served as the test data) with the procedure being repeated until all ten folds served as a test fold once. A partial correlation analysis based on 100,000 samples, in which we controlled for all the covariates from the initial robust regression reported above, revealed a significant association between the observed fluid intelligence scores and their predicted value across all 10 test folds, Spearman's *rho* of 0.11, *p* = .003 (see Fig. 5-b).

### 3.3 Fluid intelligence and affective functioning: relevance of yoked variability in FC and concrete environmental features

**Fig. 6:**
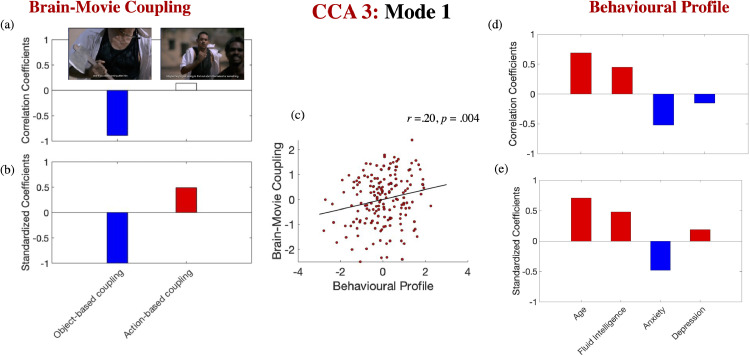
*Brain-movie coupling and cognitive-affective function in the HCP sample*. Correlation [panels a, d] and standardized coefficients [panels b, e] describing the relationship between the observed variables and the predicted value of their corresponding canonical variate across all test CCAs. The scatter plot in panel (c) describes the linear relationship between the predicted values of the two variates across all test CCAs and is based on standardized variables. In panel (a), white boxes correspond to coefficients that were not robust across all the bootstrapping samples. Gender, handedness, years of education, the summary motion metric and current negative emotional experience were introduced as covariates.

***Stronger coupling between variability in window-to-window FC and concrete environmental features predicts lower fluid intelligence and greater anxiety (HCP).*** Ten discovery CCAs were conducted to probe the relationship between age, fluid intelligence, anxiety and depression, on one hand, and coupling of FC reconfiguration with concrete environmental feature (noun vs. verb-based) changes across all 14 movies. The discovery CCAs detected one significant mode, which was validated across all test sets (*r* of 0.20, *p* = .004). This mode indicated that greater FC reconfiguration as a function of object-based, but not action-based changes, typifies anxious younger adults with lower fluid intelligence (see Fig. 6 for the correlation [panels a, d] and the standardized coefficients [panels b, e] of the behavioural and brain-movie coupling variables on their corresponding canonical variate across all test CCAs, as well as the scatter plot describing the linear association between the behavioural variate and the brain-object-based movie coupling across all the “test” folds [panel c]).

### 3.4 Brain network interactions underpinning FC variability in response to concrete environmental fluctuations and abstract event boundaries

#### 3.4.1 Informational flow across DMN ROIs predicts event boundary-based FC reconfiguration, independent of age and average window-to-window FC reconfiguration (Cam-CAN)

**Fig. 7:**
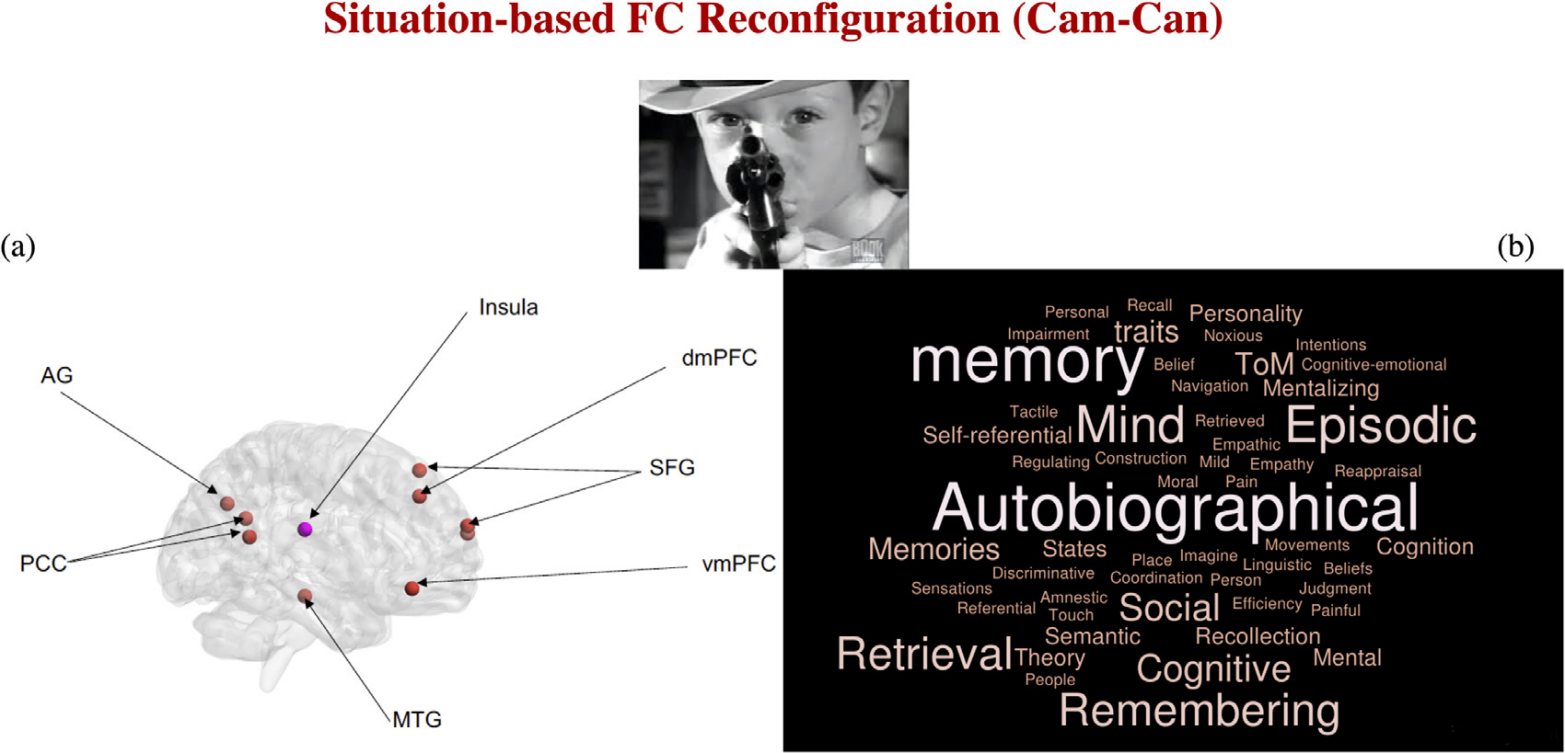
Panel (a). Results of the PLS analysis, showing the ROIs that demonstrated robust associations with situation-based FC brain reconfiguration across the lifespan. The brain regions were visualized with the BrainNet Viewer (http://www.nitrc.org/projects/bnv/) (Xia et al., 2013). ROI colours reflect Power et al.’s network assignments (orange, DMN; pink, AUD). Panel (b) Results of the Neurosynth decoding analysis based on the ROIs in panel (a). AG = angular gyrus; PCC = posterior cingulate cortex; MTG = middle temporal gyrus; SFG = superior frontal gyrus; dmPFC = dorsomedial prefrontal cortex; vmPFC = ventromedial prefrontal cortex.

The behavioral PLS analysis identified a single ROI-participation LV (*p* = .0005) which was significantly linked to reorganization in response to event boundaries, independent of age and window-to-window FC reconfiguration levels (*r* = 0.25, 99% CI= [.25; 0.45]). Ten ROIs, all but one in the DMN, made a reliable contribution (absolute value BSR > 3) to this LV (insula, angular gyrus [AG], middle temporal gyrus [MTG], posterior cingulate cortex [PCC], superior frontal gyrus [SFG], dorsomedial and ventromedial prefrontal cortex [dmPFC, vmPFC], see Fig. 7-a).

Subsequently, we conducted a decoding analysis in Neurosynth (Yarkoni et al., 2011), focused on the central voxel within each of the ROIs robustly linked by PLS to FC reconfiguration in response to event boundaries, in order to shed some light on their previously documented functional associations. As can be seen in Fig. 7-b, the analysis revealed that the strongest z-score-based (Neurosynth z-scores > 4) associations were with “memory”, “autobiographical”, “episodic”, “retrieval”, “mind” and “remembering”. These decoding results are compatible with the interpretation that brain sensitivity to more abstract event boundaries is uniquely associated with greater functional integration of ROIs that are relevant to the formation of ongoing event representations and play a key role in internally guided mnemonic processes (Honey et al., 2017; Stawarczyk, Bezdek, and Zacks, 2019).

Speaking to the specificity of these effects, a second (control) behavioural PLS analysis revealed that informational flow across a distinct subset of DMN, VIS and FPC ROIs predicts window-to-window FC reconfiguration, independent of age and event boundary-based FC reconfiguration level (see “A subset of DMN, VIS and FPC ROIs predicts window-to-window reconfiguration, independent of age and event boundary-based reconfiguration level (Cam-CAN)” in the Supplementary Materials).

#### 3.4.2 Informational flow across a subset of the DMN ROIs linked to event boundary-based FC reconfiguration predicts coupling between variability in window-to-window FC and concrete environmental features (HCP)

We conducted ten discovery CCAs, probing the link between brain-environment coupling based on object-based variations and participation of the ten ROIs uniquely linked in the Cam-CAN to FC reconfiguration evoked by more abstract event boundaries. Because we were interested specifically in brain-environment coupling with respect to object-related variations (due to its relevance to cognitive-affective functioning, as shown in CCA 3 [Fig. 6]), we regressed out from the brain-index not only global window-to-window FC reconfiguration (as in prior analyses), but also brain-environment coupling based on action/verb-related changes. Age was introduced in this analysis to probe whether the link between brain-environment coupling and the narrative ROI participation profile varies with age (in the Cam-CAN, the ROI participation profiles were shown to contribute to high-level event boundary-based reconfiguration irrespective of age, but it was unclear whether the same would be true with to lower-level featural fluctuations).

**Fig. 8:**
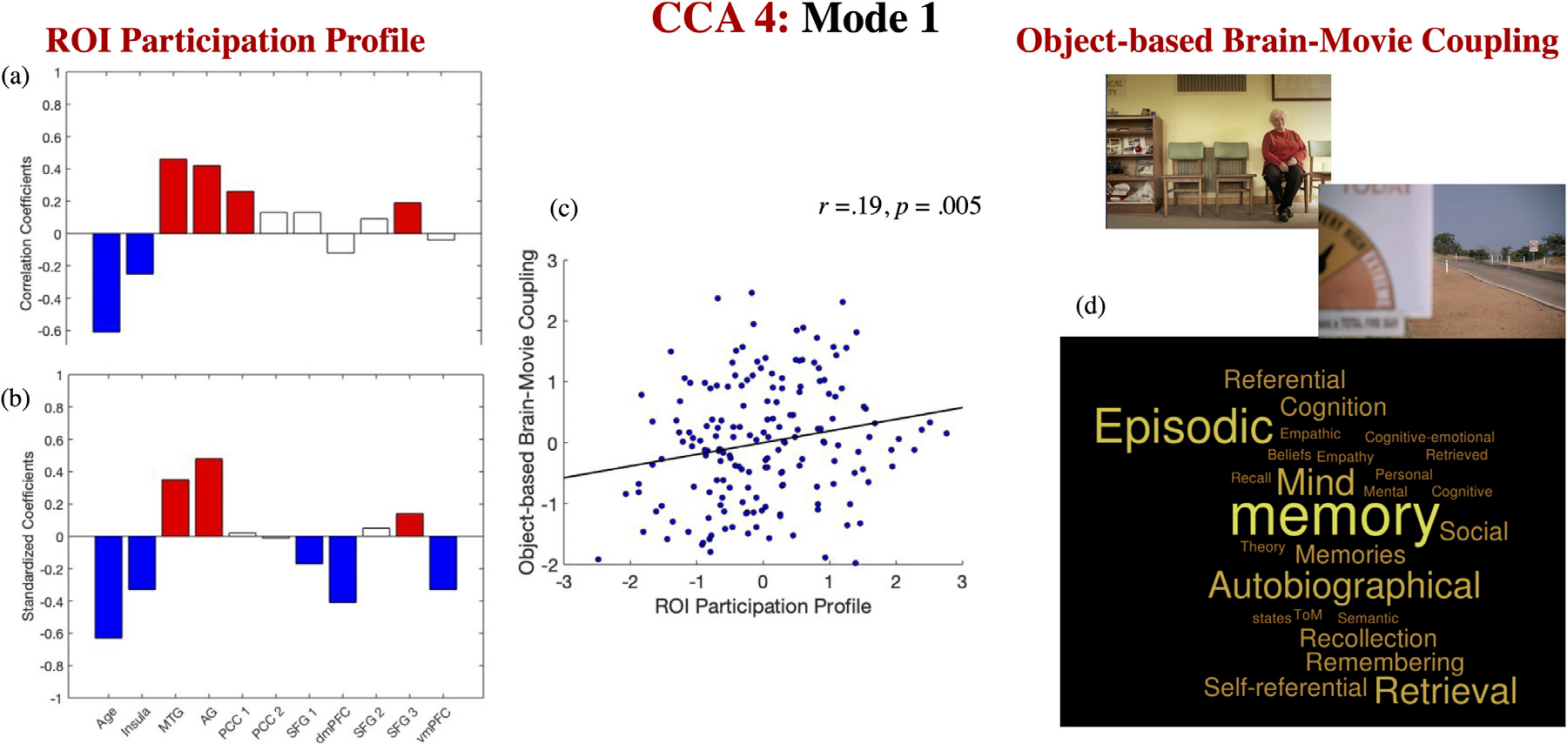
*The ROI participation profile linked to brain-object-based movie coupling.* Correlation [panel a] and standardised coefficients [panel b] describing the relationship between the observed variables and the predicted value of their corresponding canonical variate across all test CCAs. The scatter plot in panel (c) describes the linear relationship between the predicted values of the two variates across all test CCAs and it is based on standardised variables. Panel (d) presents the results of the Neurosynth decoding analysis based on the ROIs with robust loadings in panel (a). In panels (a) and (b), white boxes correspond to coefficients that were not robust across all the bootstrapping samples. Gender, handedness, years of education and the summary motion metric were introduced as covariates.

One significant mode emerged from the discovery CCAs, which was replicated across all test sets (*r* of 0.19, *p* = .005). This mode indicated that stronger brain-environment coupling (with respect to object-based fluctuations) was associated with greater participation across most of the Cam-CAN ROIs at younger ages, but particularly the medial temporal and parietal ROIs, which, based on Neurosynth decoding, were most relevant to “memory”, “autographical”, “episodic” and “retrieval” (see Fig. 8 for the correlation [panel a] and the standardized coefficients [panel b] of the ROI participation variables on their corresponding canonical variate across all test CCAs, the scatter plot describing the linear association between the ROI participation variate and the brain-object-based movie coupling across all the “test” folds [panel c], as well as the results of the Neurosynth decoding analysis [panel d] constrained to the ROIs that showed robust correlations with the extracted covariate in panel [a]).

## 4 Discussion

Extending prior literature on the role of brain signal variability in fostering a more differentiated and flexible response to the environment (e.g., Garrett et al., 2013a,b, 2015, 2020; Grady and Garrett, 2018), we provide novel evidence that the adaptiveness of both moment-to-moment and event boundary-based fluctuations in whole-brain FC patterns varies as a function of the underlying network communication profiles. We further demonstrate that enhanced brain-environment alignment with respect to concrete featural fluctuations is stronger at younger ages, but associated with adverse implications for both cognitive and affective functioning (i.e., fluid intelligence, depression/anxiety). Finally, we document the network integration profiles that link functional brain reconfiguration at multiple timescales and are, thus, likely to be key to understanding the dynamics behind typical and atypical variations in event processing.

### 4.1 Fluid intelligence across adulthood: relevance of FC variability and associated profiles of functional network interactions

Using a naturalistic, dynamic cognition paradigm in an adult lifespan sample, we demonstrate that both moment-to-moment and event boundary-based FC variability increase with age. However, contrary to the hypothesis that such increase would reflect neural instability and, thus, be negatively associated with fluid intelligence, we observed no significant association between fluid intelligence and either type of FC variability.

Instead, the adaptiveness of FC reconfiguration appeared to hinge on the associated patterns of whole-brain network participation. Specifically, in line with our hypotheses and extant theories, superior higher fluid intelligence, primarily indexed as abstract reasoning ability, was linked to greater functional integration of networks implicated in vigilance and control maintenance (CON), as well as environmentally driven attention (VAN) and behavioural regulation (SAL) (see Fig. 4-a, Barbey, 2018; Corbetta and Shulman, 2002; Duncan et al., 2020; Sadaghiani and D'Esposito, 2015; Seeley et al., 2007; Sridharan et al., 2008). As predicted, this network communication profile tended to typify FC reconfiguration during younger adulthood. These results thus dovetail nicely with findings from the literature on brain signal variability, thereby implying that the beneficial consequences of both signal and FC variability are likely to involve neural tuning to the dynamics of the external world, an ability that seems to decline with advancing age (Garrett et al., 2013a, 2020; Grady and Garrett, 2018).

Complementarily, the participation profile linked to FC variability during older adulthood was typified by reduced CON, VAN, and SAL participation and, instead, as we predicted, it reflected most strongly the diverse interactions of the networks involved in self-guided cognition (DMN) and top-down control (FPC), in particular, but also of those implicated in goal-directed attention (DAN) and visual processing (Andrews-Hanna et al., 2014; Corbetta et al., 2000; Spreng et al., 2010) (see Fig. 3-a). Our finding that FC reconfiguration in older adulthood depends on neural communication pathways grounded in the DMN and FPC complements current theories of cognitive aging, which posit that age-related declines in the ability to engage strategically with the external environment in the here-and-now are compensated by drawing on accumulated world knowledge (Li et al., 2004; Spreng and Turner, 2019). This age-related semanticisation, stemming from progressively stronger functional coupling between the DMN and the FPC (Turner and Spreng, 2015), helps preserve task performance in contexts where prior knowledge is relevant (Umanath and Marsh, 2014). One such context may be event segmentation, where performance preservation with aging may be due to increasing reliance on semantic knowledge (rather than perceptual representations) during event perception (Radvansky and Dijkstra 2007). This conjecture is compatible with recent findings of aging-related activity reductions in canonical episodic memory areas, but not schematic/conceptual processing areas, in response to narrative event boundaries observed in the Cam-CAN sample, an effect that emerges despite the lack of age-related differences in behavioural event segmentation (Reagh et al., 2020). Importantly, though, we did not find a significant association between crystallized intelligence (i.e., semantic knowledge) and the participation profile typifying FC variability in older age. Hence, rather than reflecting aging-related semanticisation, this network profile may simply indicate aging-related deficits in DMN disengagement, which may underpin its greater functional integration, as observed here (Samu et al., 2017).

### 4.2 Fluid intelligence and affective functioning: relevance of yoked FC and concrete environmental variability

Complementing findings on the adaptiveness of modulating brain signal variability based on environmental complexity (e.g., Garrett et al., 2020), our study provided novel insights into the adverse functional implications linked to coupling in the dynamics of whole-brain FC and concrete environmental features. Specifically, prior evidence indicates that anxiety and depression are associated with divergent processing biases, which impact event perception (Belzung et al., 2015; Bishop et al., 2004; Brewin et al., 2010; Petrican et al., 2015; Sherrill et al., 2019). Accordingly and in line with our hypothesis, we found that subclinical anxiety was linked to increased brain-environment alignment, implying greater tuning into the dynamics of the external perceptual world (Fig. 6-a, b, d, e), while an opposite tendency, consistent with attentional disengagement from concrete environmental dynamics in the here-and-now, was observed for subclinical depression (Fig. 6-e).

Contrary to our hypothesis, coupling between window-to-window FC and concrete featural fluctuations was associated with poorer fluid intelligence. This implies that, instead of reflecting more differentiated information processing (cf. brain signal variability, Garrett et al., 2020), FC alignment with concrete environmental dynamics is likely indicative of preferential reliance on sensory-bound, rather than more abstract mental representations, a cognitive predisposition which tends to prevent successful strategic processing and has been linked to both state and trait anxiety (Hermans et al., 2014; Mathews, Yiend, & Lawrence, 2006; Moser et al., 2012; Sylverster et al., 2012).

Importantly, associations with cognitive and affective functioning indices were specific to coupling between FC and object/spatial layout fluctuations. The specificity of these effects is unsurprising because noun-based descriptors are likely to capture best concrete features of the external environment. Nonetheless, since action-based changes play a key role in event segmentation processes from childhood onwards (Levine et al., 2019; Magliano and Zacks, 2011; Swallow et al., 2018), further investigation of their relevance to brain-environment alignment and adaptive functioning is certainly warranted.

### 4.3 Brain network interactions underpinning FC variability in response to concrete environmental fluctuations and abstract event boundaries

Our study also contributed novel evidence on the overlapping network communication profiles underlying brain-environment alignment with respect to changes in both concrete environmental features and in the more abstract relational representation of an ongoing situation (i.e., event boundaries). In both cases, brain-environment coupling was associated with greater whole-brain informational flow (i.e., participation) in a subset of canonical DMN ROIs, including the left AG, left MTG, PCC and left SFG, the majority of which had been implicated in event memory reactivation (Chen et al., 2017). The AG and PCC play key roles in recollection (Ranganath and Ritchey, 2012; Ritchey and Cooper, 2020; Rugg and Vilberg, 2013) and have been widely implicated in the integration of multimodal information at longer timescales, thereby supporting the creation of the so-called “event models”(Bonnici et al., 2016; J. Chen et al., 2016; Radvansky and Zacks, 2017; Stawarcyzk et al., 2019; Yazar et al., 2017). The left AG, in particular, plays a causal role in episodic context creation during perception by acting as an online buffer for combining past and currently presented information (Branzi et al., 2019; Humphreys and Lambon Ralph, 2015). Expanding this literature, we document the role of AG in integrating information across the whole brain during event perception in order to align the external environmental and internal neural dynamics, and, potentially regulate the switch between internally and externally-based processing modes (Brandman et al., 2021).

The left MTG demonstrated a similarly robust association with functional brain reconfiguration triggered by both concrete featural and more abstract relational changes in the external environment. Like AG, this region underpins updating of semantic features related to the present context (Branzi et al., 2020), while uniquely partaking into the controlled retrieval of semantic information (Hoffman et al., 2018). The MTG and AG could, plausibly, make complementary contributions to the creation and updating of event representations (Kurby & Zacks, 2008). Specifically, the AG-centered participation profile may provide the episodic perceptual detail from which a strong sense of sensory vividness and grounding in the here-and-now stem (Ramanan et al., 2018). Complementarily, the MTG-linked participation profile may support continual updating of the underlying semantic structure based on the influx of environmental information and controlled retrieval of already stored world knowledge, thereby synching one's mental representation of the immediate environment with the ongoing experience (Hoffmann et al., 2018; Zacks, 2020).

### 4.4 Limitations and future directions

Future studies are warranted to address limitations of our present research. First, use of a larger battery of movies (as in HCP), covering diverse artistic interests and production dates, with a strong narrative plot that allows reliable extraction of narrative event boundaries (as in Cam-CAN), is needed to characterize hierarchical event perception dynamics. Second, inclusion of a full lifespan sample, as well as complementary cross-sectional/longitudinal designs may elucidate the role of brain-environment entrainment during developmental stages characterised by distinct learning needs (Baldwin and Kosie, 2020; Li et al., 2004). Third, although behavioural event segmentation is largely preserved in healthy aging (Kurby and Zacks, 2018; Reagh et al., 2020; Sargent et al., 2013), future studies probing the link between event segmentation performance and its neural substrates across the lifespan would be critical in furthering our understanding of developmental differences in information processing. Fourth, research employing alternate methods for estimating dynamic FC reconfiguration would elucidate the boundary conditions of the effects herein documented (Gonzalez-Castillo and Bandettini, 2018; Iraji et al., 2020). Fifth, our present study was based on two datasets acquired at different sites, with distinct scanning parameters and magnetic field strength. A similar approach has been adopted before (e.g., Ben-Yakov and Henson, 2018) and we have done our best to augment the comparability of the two datasets in terms of preprocessing and spatio-temporal resolution of the analyses. Nonetheless, future studies using data acquired on the same scanner could provide additional insights into our presently reported effects.

In sum, we demonstrate that the adaptiveness of dynamic FC reconfiguration during naturalistic event cognition varies based on the associated patterns of network interaction. Specifically, similar to brain signal variability, the adaptiveness of FC variability seems to hinge on its relevance to enhanced processing of the external world, which tends to decline with advancing age. Complementing the literature on environmental modulation of brain signal variability, we further show that brain-environment alignment with respect to concrete, featural fluctuations is a potential indicator of perceptually bound processing, which is more pronounced in younger adulthood and carries adverse implications not only for affective functioning, but also for strategic cognitive engagement with novel environments. Finally, we characterize network communication profiles which link event segmentation processes across multiple timescales by providing episodic and semantic scaffolding to context formation during perception, as well as during the subsequent retrieval of these event representations.*Acknowledgments*. K.S.G. and A.D.L. were funded by grants from the Medical Research

Council (MR/N01233X/1) and a Wellcome Trust Strategic Award (104,943/Z/14/Z). For the purpose of Open Access, the authors have applied a CC BY public copyright licence to any Author Accepted Manuscript version arising from this submission. Data were provided by the Cambridge Centre for Ageing and Neuroscience (Principal Investigator: Lorraine K. Tyler; funders: the Biotechnology and Biological Sciences Research Council, the Medical Research Council Cognition & Brain Sciences Unit and the European Union Horizon 2020 LifeBrain project) and the Human Connectome Project, WU-Minn Consortium (Principal Investigators: David Van Essen and Kamil Ugurbil; 1U54MH091657; funders: the 16 NIH Institutes and Centers that support the NIH Blueprint for Neuroscience Research and the McDonnell Center for Systems Neuroscience at Washington University).

## Data statement

The raw data are available from https://camcan-archive.mrc-cbu.cam.ac.uk/dataaccess/ (Cam-CAN) and https://db.humanconnectome.org/app/template/Login.vm;jsessionid=90091F006B15D5FA1D1D-9C3ED2D465DD (HCP) upon completion of the relevant data use agreements.

## Code availability

We used already existing code, as specified in the main text with links for free download.

## CRediT authorship contribution statement

**Raluca Petrican:** Conceptualization, Methodology, Formal analysis, Data curation, Visualization, Writing - original draft. **Kim S. Graham:** Writing - review & editing, Funding acquisition. **Andrew D. Lawrence:** Conceptualization, Writing - review & editing, Funding acquisition.

## Declaration of Competing Interest

The authors declare no competing interests.

## Appendix Supplementary materials

Image, application 1

Image, application 2
