# Supplementary material for: Brain-environment alignment during movie watching predicts fluid intelligence and affective function in adulthood
Source: Neuroimage. 2021 Sep;238:118177. doi: 10.1016/j.neuroimage.2021.118177 (PMC8350144; doi:10.1016/j.neuroimage.2021.118177)
Supplement: Supplementary file 1 [file mmc1.docx]

**Supplementary Materials**

**Method**

**Fit of the Power atlas to the Cam-Can dataset.** To evaluate the homogeneity of the Power ROIs across the Cam-Can adult lifespan sample (cf. Iraji et al., 2020), we used an approach that is conceptually similar to those recently used in the literature (Gordon et al., 2016; Siegel et al., 2016). Specifically, we used the CONN toolbox to compute the radial similarity contrast (RSC) for each voxel in the Power atlas. As implemented in CONN, the RSC reflects the amount of similarity in whole brain connectivity patterns between a voxel and its neighbours in each of the three space directions (x,y,z), and is thus a 3 dimensional construct (see also Kim et al., 2010). If a node is functionally homogenous, then the RSC of its voxels should be relatively similar (i.e., across the entire ROI, there should be a consistent degree of similarity among the component voxels’ whole brain connectivity patterns). To test this hypothesis, for each participant, we conducted a principal components analysis of the RSC of the 81 voxels within each ROI (i.e., for each participant, one ROI constituted one case, whereas the RSC of one voxel within a given ROI constituted a variable). As a measure of similarity among the RSC of all voxels within a node across all nodes, we took the percent of variance explained by the first component extracted through the analysis just described (see also Gordon et al., 2016; Siegel et al., 2016). Because, as previously mentioned, RSC is a three-dimensional vector, this set of analyses yielded, for each participant, three indices of average similarity in global functional connectivity patterns among all voxels within a given ROI. The three indices were significantly positively correlated across participants (Spearman’s *rho*s from .17 to .24, all *p*s < .0001), which is why we averaged them to create a summary measure of ROI functional homogeneity. A correlational analysis, based on 100,000 permutation samples, revealed a modest, but significant negative relationship between age and the average similarity in global functional connectivity patterns among the component voxels of the Power ROIs (Spearman’s *rho* of -.21, *p* = 10^-5^). As expected, given that the Power ROIs were validated on a sample corresponding in age to the first decade of the Cam-Can sample, a second robust regression analysis revealed no evidence of significant quadratic age effects on the functional homogeneity of the Power ROIs (*p* > 0.56). Based on these results, the summary measure of ROI functional homogeneity was introduced as a covariate in all hypothesis testing analyses.

**Fit of the Power network assignment to the Cam-Can dataset.** To estimate the fit of Power et al.’s (2011) network assignments to the present data, we sought to replicate these authors’ procedure by using the same community detection algorithm, i.e., Infomap (Lancichinetti & Fortunato, 2009; Rosvall & Berstrom, 2008), as well as the same procedure for thresholding the ROI-to-ROI correlations in time series (see below). We ran our analyses using Infomap Online (D. Edler & M. Rosvall) available at [www.mapequation.org/infomap](http://www.mapequation.org/infomap).

Following Power et al. (2011), we conducted all our community detection analyses on group-averaged ROI-to-ROI correlation matrices. To test whether Power et al.’s (2011) network assignments provide an equally adequate fit to the present connectivity data across the lifespan, we first averaged the individuals’ ROI-to-ROI correlation matrices, obtained from CONN, separately within each of the seven decades. Next, we used the Brain Connectivity Toolbox (BCT, Rubinov & Sporns, 2010) to threshold each of the seven matrices at the same tie densities used by Power et al. (2011) for the areal graph (i.e., 2% to 10% tie density in increments of 1%). BCT was subsequently utilized to write the thresholded matrices into a Pajek *.net format and the resulting files were inputted into Infomap. Infomap was run with the option of extracting a one-level structure (i.e., no nested modules), which is the format of Power et al.’s (2011) atlas as is publicly available. Finally, we used again the BCT to compute the normalized mutual information index (NMI; range: 1 [perfect similarity] to 0 [no similarity at all]) as a measure of similarity between Power et al.’s network assignments and those outputted by Infomap for the connectivity data from each of the Cam-Can’s seven decades (Fornito, Zalesky & Bullmore, 2016). As can be seen in Figure S1, across all tie densities, the network structure from each of the seven age groups exhibited equivalent levels of similarity with that proposed by Power et al. (2011). These results thus suggest that Power et al.’s network structure provide an adequate fit to the present connectivity data across the lifespan (i.e., the NMI values were similar to those documented by Power et al. when comparing network structure across cohorts).

Use of Power et al.’s procedure was necessary to evaluate the fit of their network assignments to the Cam-Can lifespan dataset with minimal interference from the confounding effects of using different community extraction algorithms. However, thresholding of the ROI-to-ROI correlations in time series is a suboptimal strategy for investigating for individual differences (Betzel et al., 2014; Satterthwaite et al., 2015). This is why the main analyses described in our report employed the Louvain community extraction algorithm, whose use with both positive and negative correlation coefficients has been extensively researched and validated (e.g., Chen et al., 2016; Fornito et al., 2016; Rubinov & Sporns, 2011).

**Community detection*.*** Rather than being computed directly, the degree to which a network can be fragmented into well-delineated and non-overlapping communities or modules is estimated using optimization algorithms, which sacrifice some degree of accuracy for processing speed (Fornito et al., 2016; Rubinov & Sporns, 2010). Here, the optimal whole-brain division into non-overlapping communities was estimated using a Louvain community detection algorithm implemented in the BCT. This algorithm partitions a network into non-overlapping groups of nodes with the goal of maximizing an objective modularity quality function, Q (Betzel & Bassett, 2017; Rubinov & Sporns, 2011; Sporns & Betzel, 2016). There are multiple strategies for estimating community structure based on sliding window data, as was the case of our movie viewing data. Specifically, multilayer modularity algorithms (Bassett et al., 2011; Braun et al., 2015; Mucha et al., 2010) can provide important insights into community dynamics at multiple time scales. Nonetheless, such algorithms require estimation of additional free parameters (e.g., the temporal coupling parameter between two adjacent temporal windows). Since we feared that estimation of the temporal coupling parameter could act as a potential confound when comparing connectivity results across the multiple samples included in the analysis, we used the same procedure to estimate community structure independently in each sliding window (see also Chen et al., 2016), as described below.

For signed networks, such as the ones investigated in our study, optimization of the Q function can be achieved by either placing equal weight on maximizing positive within-module connections and minimizing negative within-module connections or by putting a premium on maximizing positive connections, which have been argued to be of greater biological significance (Fornito et al., 2016; Rubinov & Sporns, 2011). Although we verified that all the reported results emerge with either formula, for the sake of simplicity and because we agree with their argument regarding the greater importance of positive weights in determining node grouping into communities, we report here the results based on Rubinov and Sporns’s modularity formula (cf. Chen et al., 2016; Rubinov & Sporns, 2011). In this formulation, the contribution of positive weights to Q is not affected by the presence of negative weights in the network, whereas the contribution of negative weights to Q decreases with an increase in positive weights.

The adapted modularity function Q^*^, proposed by Rubinov and Sporns (2011) is written as

Q^*^ = ,


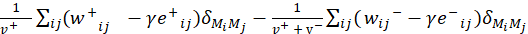

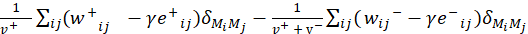


where δ_MiMj_ = 1 if nodes i and j are in the same module and δ_MiMj_ = 0 otherwise; v^+^ and v^-^ constitute the sum of all positive (w^+^) and all negative (w^-^) weights in the network, respectively; w^±^_ij_ represent the actual within-module positive or negative connection weights with w^±^ $\in(0,1]$; γ is a resolution parameter determining the size of the identified modules; e^±^_ij_ is the within-module connection strength expected by chance and defined, for each node-to-node (i,j) connection as e^±^_ij_ = $\frac{s_{i}^{\pm}s_{j}^{\pm}}{v^{\pm}}$, with s^±^_i_ and s^±^_j_ being the sum of all positive or all negative connection weights of node i and j, respectively, while v^±^ is the sum of all positive or all negative connection weights in the network.

To account for the near degeneracy of the modularity landscape (i.e., many near-optimal ways of partitioning a network into non-overlapping communities, Good et al., 2010) and for changes in community structure due to variations in the estimation parameters, the community detection algorithm was each initiated 100 times for three values of the spatial resolution parameter, centered around the default value of 1 (cf. Betzel & Bassett, 2017; Braun et al., 2015; Chen et al., 2016). Based on the results of these analyses, run separately for each of the three spatial resolution values, a consensus partition (i.e., whole-brain division into constituent communities) was estimated for each participant in each movie window (cf. Bassett et al., 2013; Lancichinetti & Fortunato, 2012).

**Results**

**A subset of DMN, VIS and FPC ROIs predicts window-to-window FC reconfiguration, independent of age and situation-based FC reconfiguration level (Cam-Can).** A second behavioural PLS analysis identified one ROI participation LV (*p* = .0002) which was significantly linked to window-to-window reorganization, independent of age and narrative context-based brain reconfiguration levels (*r* = .26, 99% CI= [.26; .46]). Nine ROIs, encompassing the DMN (dmPFC, SFG), VIS (fusiform gyrus [FG], inferior occipital gyrus [IOG]), and FPC (inferior parietal lobule [IPL], medial frontal gyrus [MFG]) made a reliable contribution (absolute value BSR > 3) to this LV (see Figure S2-a).

A decoding analysis in Neurosynth, similar to the one reported in the main text for narrative context-based reconfiguration, revealed that the ROIs linked to window-to-window brain reconfiguration showed strongest functional associations with “visual”, “memory”, “objects”, “language”, “social”, “mind”, and “retrieval” (see Figure S2-b). This functional web was quite distinct from the one observed for the ROIs uniquely associated with context-based reconfiguration.

**Cross-validation of the behavioural PLS analyses.** To test the robustness of our PLS results involving event boundary-based FC reconfiguration (behavioural PLS analysis 1, reported in the main text) and window-to-window FC reconfiguration (behavioural PLS analysis 2, reported above), we implemented a ten-fold cross-validation procedure similar to the one used in CCAs. Specifically, data were broken down into 10 folds, behavioural PLS was run on 9 folds of data with the tenth being used as a test fold, with the procedure being repeated until all data served as a test fold once. For each of the two behavioural PLS analyses, the corresponding brain LV was reconstructed in the test folds by using the ROI saliences from the discovery folds (that did not include the respective test fold). 100,000 bootstrapping samples were generated using bootci in Matlab to evaluate the correspondence between the brain LVs reconstructed in the test folds and the originally extracted LVs (as reported in the main text), as well as the corresponding “behavioural” variables. For both PLS analyses, the brain LV reconstructed in the test folds showed almost complete overlap with the initially extracted brain LV, *r*s of .97, 99% CI= [.96; .97], and .98 99% CI= [.97; .98]. As expected, the brain LV reconstructed in the test folds also demonstrated a significant correlation with the associated “behavioural” variable, i.e., event boundary-based FC reconfiguration (controlling for age and window-to-window FC reconfiguration), *r* of .13, 99% CI= [.02; .24], and window-to-window FC reconfiguration (controlling for age and event boundary-based FC reconfiguration), *r* of .18, 99% CI= [.07; .28].

**Reliability** **of the behavioural PLS analyses.** To test the reliability of the brain LVs identified in each of the two behavioural PLS analyses conducted in the Cam-Can sample, we implemented a split-half resampling procedurewhich is part of the original PLS toolbox and assesses the reliability of the extracted LVs (Kovacevic, Abdi, Beaton, & McIntosh, 2013). Specifically, for each of the two behavioural PLS analyses, the data were randomly split into halves and the saliences from the initial PLS analysis performed on the full sample were used to compute the brain LV scores in each of the two halves (our behavioural PLS analyses were based on data from a single condition, hence we could only evaluate reliability of the brain LV). For each behavioural PLS analysis, the split-half resampling procedure was repeated 1000 times. The significance of the observed correlation in the projected brain LVs between the two halves was evaluated with permutation-based testing (5000 permutation samples). The results of the split-half resampling procedure confirmed the reliability of the brain LVs identified in the analysis involving event boundaries, *r* = .21, permutation-based *p* = .025, and window-to-window FC reconfiguration, *r* = .21, permutation-based *p* = .022.

References

Kovacevic, N., Abdi, H., Beaton, D., McIntosh, A.R. (2013). Revisiting PLS resampling: comparing significance versus reliability across range of simulations. In: New Perspectives in Partial Least Squares and Related Methods, New York, NY, USA: Springer,159–70.
