# Supplementary material for: Brain-environment alignment during movie watching predicts fluid intelligence and affective function in adulthood
Source: Neuroimage. 2021 Sep;238:118177. doi: 10.1016/j.neuroimage.2021.118177 (PMC8350144; doi:10.1016/j.neuroimage.2021.118177)
Supplement: Supplementary file 2 [file mmc2.pdf]

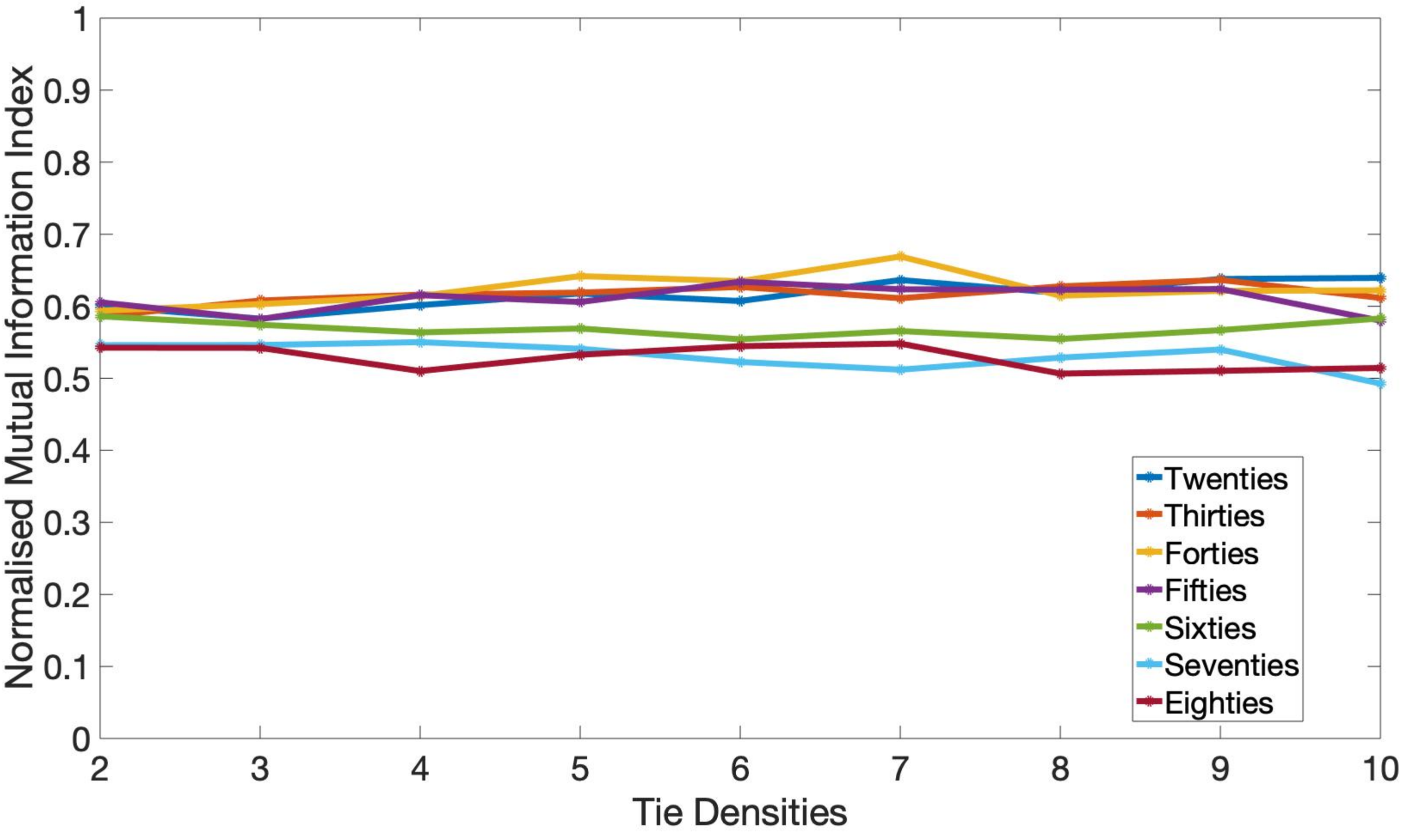

Figure S1. Network structure in each of the seven age groups of the Cam-Can sample during movie watching at of 2-10% tie densities shows equivalent levels of similarity to that reported by Power et al. (2011) across various cohorts during rest. The coloured lines show the normalised mutual information index, a highly used metric of similarity in community assignment, for each age group and tie density scrutinised.

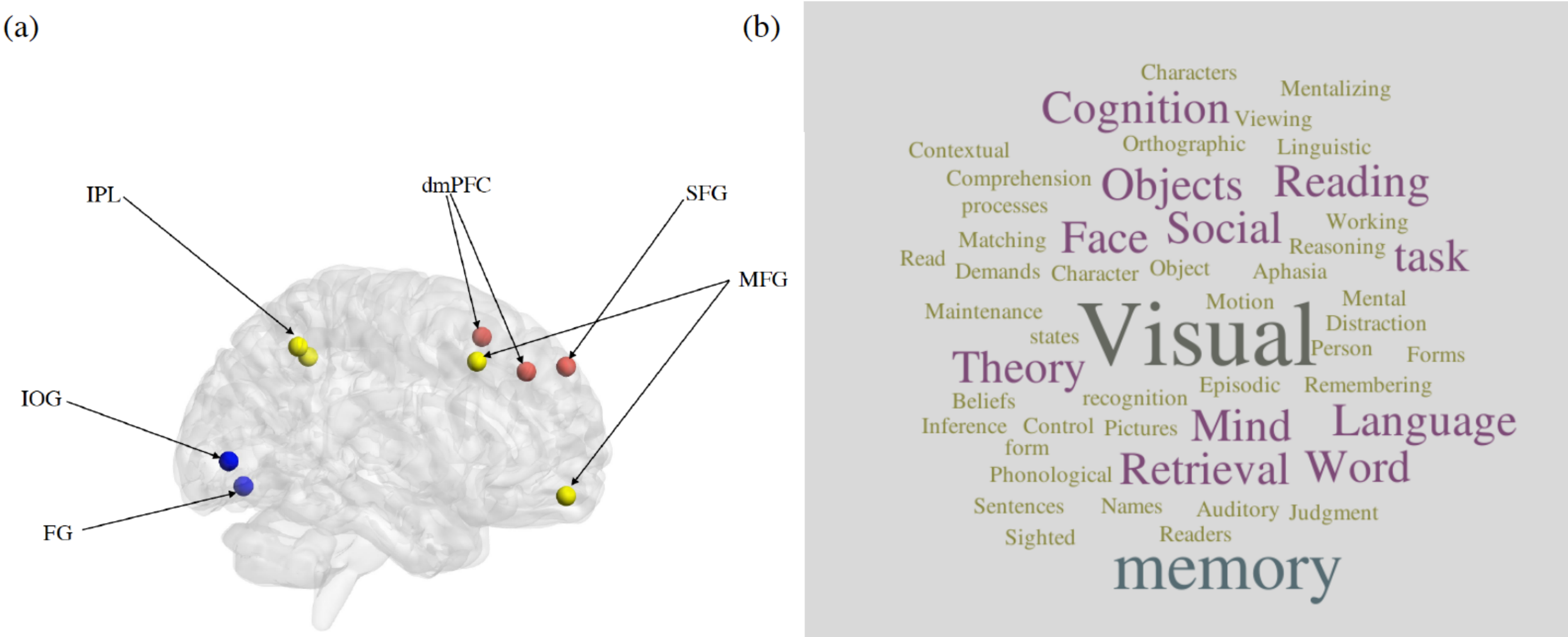

Figure S2. Panel (a). Results of the PLS analysis, showing the ROIs that demonstrated robust associations with window-to-window functional brain reconfiguration across the lifespan. The brain regions were visualized with the BrainNet Viewer (http://www.nitrc.org/projects/bnv/) (Xia et al., 2013). ROI colours reflect Power et al.'s network assignments (orange, DMN; blue, VIS; yellow, FPC). Panel (b) Results of the Neurosynth decoding analysis based on the ROIs in panel (a).
